# Phylogenomic relationships and species delimitation of *Cotoneaster* ser. *Pannosi*, ser. *Buxifolii*, and related taxa

**DOI:** 10.3389/fpls.2025.1575925

**Published:** 2025-05-20

**Authors:** Jiaxuan Huang, Sufang Chen, Kaikai Meng, Mingwan Li, Wanyi Zhao, Na Wang, Qiang Fan, Wenbo Liao

**Affiliations:** ^1^ State Key Laboratory of Biocontrol and Guangdong Provincial Key Laboratory of Plant Resources, School of Life Sciences, Sun Yat-sen University, Guangzhou, China; ^2^ Guangxi Key Laboratory of Quality and Safety Control for Subtropical Fruits, Guangxi Subtropical Crops Research Institute, Nanning, China; ^3^ College of Forestry, Henan Agricultural University, Zhengzhou, China

**Keywords:** *Cotoneaster*, phylogeny, ddRAD-seq, species delimitation, integrative taxonomy

## Abstract

Polyploidy and hybridization are prevalent phenomena within the genus *Cotoneaster*, leading to blurred species boundaries, particularly in *Cotoneaster* ser. *Pannosi* and *Cotoneaster* ser. *Buxifolii*. This study seeks to establish a robust phylogenetic framework for these series and their allied taxa to support future taxonomic revisions and investigations of hybridization–polyploidy dynamics. Population-level sampling was conducted across 43 populations located in Sichuan, Taiwan, Yunnan, Tibet (China), and Rasuwa (Nepal), including 17 species from *C.* ser. *Pannosi* and *C.* ser. *Buxifolii*, along with 10 species from closely related series. Following detailed comparisons with type specimens, six quantitative traits were measured, and 16 qualitative traits were recorded from individual specimens, followed by hierarchical clustering and principal component analyses of the combined dataset. Phylogenetic relationships were reconstructed using two datasets: 1) chloroplast genomes generated through shallow genome sequencing and 2) single-nucleotide polymorphisms (SNPs) obtained from restriction site-associated DNA sequencing (RAD-seq), complemented by genetic structure analyses. The taxonomic framework equally prioritizes nuclear clade monophyly [Shimodaira–Hasegawa approximate likelihood ratio test (SH-aLRT) ≥ 80% and ultrafast bootstrap (UFboot) ≥ 95%] and discrete genetic cluster membership (cluster assignment probability ≥ 95%) as primary delimitation criteria, complemented by morphological discontinuity (≥ 2 traits) and chloroplast phylogeny concordance. Fourteen species satisfied all criteria, corresponding to nine distinct gene pools, while the remaining 13 species displayed admixed genomic compositions and cytonuclear discordances, indicative of hybrid origins. This study identifies putative hybrid taxa and provides a foundational framework for further species delimitation, advancing future research on *Cotoneaster* systematics, natural hybridization patterns, and taxonomic revision.

## 1 Introduction

The tribe Maleae Small, comprising approximately 27 genera and 912 species, represents one of the most widely distributed and morphologically diverse taxa within the family Rosaceae Juss., predominantly occurring in temperate Northern Hemisphere regions (Wang et al., 2024). This tribe is characterized by the pome, a unique fruit type that serves as a key adaptive innovation (Potter et al., 2007). Economically significant members include globally cultivated fruit crops such as apples (*Malus pumila* Mill.) and pears (*Pyrus* spp.), alongside horticulturally important ornamentals like cotoneasters (*Cotoneaster* spp.) with their persistent berries, firethorns (*Pyracantha* spp.) bearing brightly colored pomes, and flowering crabs [*Malus* sp*ectabilis* (Ait.) Borkh.] valued for their showy blossoms. Extensive morphological and molecular evidence reveals pervasive hybridization patterns within and between genera, supported by congruent data from chloroplast/nuclear gene sequences and cytonuclear phylogenetic reconstructions (Liu et al., 2022; Volk et al., 2019; Liston et al., 2021). Molecular systematics indicates the tribe originated through an ancient whole-genome duplication event, likely involving hybridization between two ancestral lineages with distinct chromosomal bases followed by genome doubling (Campbell et al., 2007; Hodel et al., 2022; Sun et al., 2024). Contemporary polyploidization remains evolutionarily significant, with multiple occurrences of tetraploidy and other ploidy variations documented across the tribe (Burgess et al., 2014; Dickinson et al., 2007; Dickinson, 2018; Macková, 2020). This genomic dynamism is further amplified by widespread apomixis, which facilitates rapid reproductive isolation between ploidy variants and hybrid lineages, thereby accelerating speciation processes (Campbell and Dickinson, 1990; Campbell et al., 1991; Cushman et al., 2017). The frequent occurrence of hybridization, polyploidization, and apomixis have collectively obscured species boundaries and generated complex phylogenetic discordances, creating persistent challenges for taxonomic delineation and evolutionary reconstructions.

*Cotoneaster* Medik. represents one of the most quintessential examples within the tribe Maleae. This genus is extensively distributed across the Northern Hemisphere, encompassing all of Europe, North Africa, and the temperate regions of Asia (excluding Japan), with its diversity hotspot centered in the Himalayas and the mountainous regions surrounding Yunnan and Sichuan provinces in China (Lu and Brach, 2003; Fryer and Hylmö, 2009). Cytogenetic studies demonstrate a polyploid series across the genus: 70%–76% of species are tetraploid (2n = 68), 9%–15% are triploid (2n = 51), and 10%–15% are diploid (2n = 34), with rare occurrences of pentaploid (2n = 85) and hexaploid (2n = 102) cytotypes (Fryer and Hylmö, 2009; Rothleutner et al., 2016). The taxonomy of *Cotoneaster* has been widely debated due to differing opinions on critical morphological traits, resulting in varying species numbers. Koehne (1893) foundational system classified *Cotoneaster* into two subgenera, *Chaenopetalum* (Koehne) G. Klotz (six species) and *Cotoneaster* (seven species), based on floral traits like the angle of petals opening. Subsequent revisions introduced competing frameworks: Flinck and Hylmö (1966) organized 174 species into a hierarchical system of two sections, four subsections, and 24 series using stamen and pyrene counts; Yu et al. (1974) alternatively proposed three sections (*Uniflos*, *Cotoneaster*, and *Densiflos*) defined by inflorescence dimensions. Lu and Brach (2003) adopted a non-hierarchical approach, circumscribing approximately 90 species (including 59 in China with 37 endemics) through multivariate analysis of inflorescence structure, petal coloration, and foliar morphology. The most recent synthesis by Fryer and Hylmö (2009) integrates growth habit, trichome characteristics, shoot phenology, and reproductive morphology to classify 460 taxa under an expanded system of two subgenera, 11 sections, and 37 series, representing the current taxonomic consensus.

Recent phylogenomic analyses by Meng et al. (2021), integrating chloroplast genomes and 204 low-copy nuclear genes across 69 *Cotoneaster* species (72 accessions), resolve the genus into two principal clades (Co and Ch) corresponding to the subgeneric divisions *Cotoneaster* and *Chaenopetalum* sensu Fryer and Hylmö (2009). The Co clade exhibits prolonged sequential flowering in cymose inflorescences with predominantly erect red petals (occasionally semi-spreading or pink), although lacking internal phylogenetic resolution. Conversely, the Ch clade demonstrates synchronous cymose flowering with fully expanded white petals (rarely pink), subdivided into three well-supported subclades. These molecular phylogenies reveal non-monophyly of most traditionally defined series and pervasive cytonuclear discordance indicative of recurrent hybridization and incomplete lineage sorting during the genus’ radiation. Particular interest surrounds the monophyletic Ch-E–Ch-I subclade within the Ch lineage, which predominantly aggregates taxa from series *Pannosi* (P) and *Buxifolii* (B) sensu Fryer and Hylmö (2009), including phylogenetically entangled species such as *Cotoneaster dammeri* subsp. *songmingensis* C. Y. Wu & L.-H. Zhou, *Cotoneaster morrisonensis* Hayata, and *Cotoneaster marginatus* (Loudon) Schltdl. Chloroplast phylogenies further demonstrate a cohesive grouping of series *Pannosi* and *Buxifolii* with *Cotoneaster conspicuus* Comber ex Marquand (excluding *Cotoneaster glaucophyllus* Franch. and infraspecific taxa). Notable cytonuclear incongruence emerges in *Cotoneaster buxifolius* Wall. ex Lindl, where nuclear data position it basally within the Co clade, while chloroplast evidence nests it within series *Pannosi*–*Buxifolii* (**Supplementary Figure S1**). However, comprehensive evaluation of species boundaries and interspecific gene flow remains constrained by sparse taxon sampling (one to three– accessions per species) across this hyperdiverse genus.

**Figure 1:**
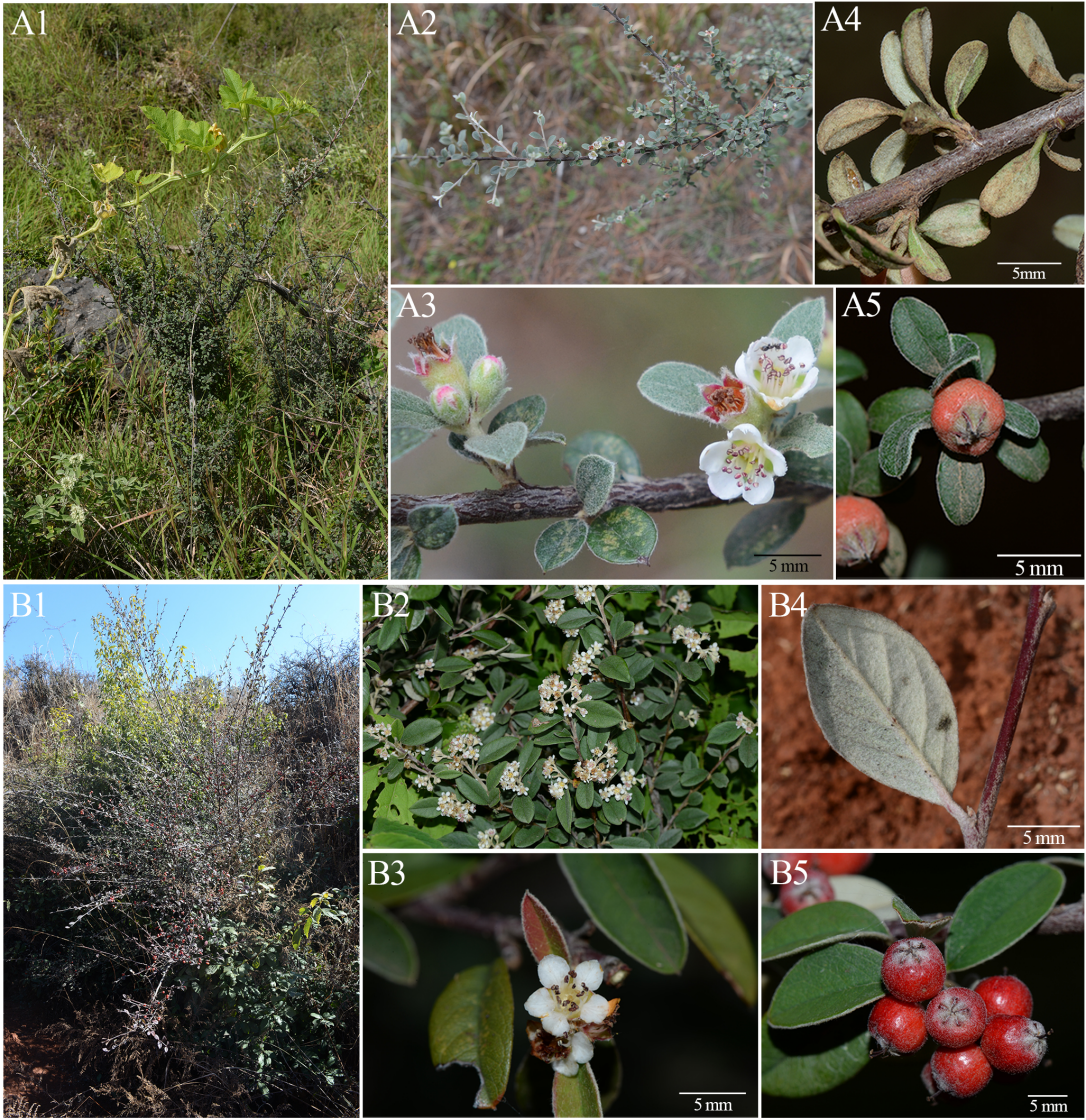
Morphological comparisons between *Cotoneaster argenteus* and *Cotoneaster pannosus* in growth habit, leaf morphology, indumentum characteristics, inflorescence, and fruits. **(A)** *C*. *argenteus*. **(A1)** Dwarf shrub (height: 0.5–1 m). **(A2)** Fertile branch with spiral branchlets. **(A3)** Flower bearing ascending petals. **(A4)** Abaxial leaf surface with slowly deciduous indumentum. **(A5)** Small globose fruit (diameter: 3.5–5 mm). **(B)** *C*. *pannosus*. **(B1)** Shrub with 1–5-m height. **(B2)** Fertile branch bearing inflorescence. **(B3)** Flower with spreading petals. **(B4)** Abaxial leaf surface retaining persistent indumentum. **(B5)** Larger fruits (diameter: 8–9 mm).

The advent of high-throughput genomic methods, particularly restriction site-associated DNA sequencing (RAD-seq), has revolutionized phylogenetic reconstruction in taxonomically complex genera such as *Diospyros* L. and *Salix* L., enhancing phylogenetic resolution and providing insights into hybridization events (Wagner et al., 2020; Linan et al., 2021). Recurrent polyploidization and hybridization events have systematically obscured species boundaries, as evidenced by whole-genome analyses revealing intricate reticulate evolution in *Helianthus* L (Renaut et al., 2014). Contemporary species delimitation frameworks emphasize integrative approaches combining genomic, morphological, and ecological data, as proposed by Liu (2016) and successfully implemented across diverse organismal groups (Ely et al., 2018; Li et al., 2019; Maharachchikumbura et al., 2021; Anjos et al., 2020; Prebus, 2021). These methodologies hold particular urgency for *Cotoneaster* given its exceptional polyploid prevalence (70%–76% tetraploids) and widespread morphological convergence, with series *Pannosi* (P) and *Buxifolii* (B) serving as exemplary case studies (Fryer and Hylmö, 2009). These Hengduan–Himalayan endemic series (15 and nine species) exhibit remarkable ecological versatility: cold/drought tolerance, avian-frugivore mutualisms, and horticultural value exemplified by *Cotoneaster lacteus* W. W. Smith and *Cotoneaster coriaceus* Franch (Fryer and Hylmö, 2009). Morphologically, ser. *P* demonstrates elliptic laminas (6–70 mm) with densely tomentose abaxial surfaces and compact cymes, whereas ser. *B* comprises dwarf shrubs with reduced foliage (3–17 mm) and 1–25-flowered inflorescences. Subtle yet consistent differentiation persists in lamina shape, indumentum density, and the number of flowers per inflorescence (**Figure 1**). Zhou and Wu (2001) multidisciplinary revision of ser. *B* through cytological analysis of type specimens identified four morphologically diagnosable species characterized by erect growth, persistent tomentum on foliar abaxial surfaces/calyces, and typically two pyrenes. However, persistent diagnostic ambiguities arise from trait overlapping and extensive hybrid zones in sympatric populations (Li et al., 2014; Li et al., 2017), underscoring the necessity of genomic-scale data to resolve these evolutionary dynamic lineages.

This investigation focuses on resolving the phylogenetic relationships and species delimitation within the Ch-E–Ch-I subclade of the Ch lineage encompassing ser. *P*, ser. *B*, and allied taxa through integrated morphometric and population genomic approaches (**Supplementary Figure S1**). We collectively refer to the species within the Ch-E–Ch-I subclade, along with other species in ser. *P* and ser. *B*, as a morphologically similar yet phylogenetically ambiguous complex. Given that *C. buxifolius* is the earliest described species in this group, we designate this complex as the *C. buxifolius* complex. We sampled a total of 30 populations representing 16 recognized taxa within this complex. We meticulously recorded morphological traits for traditional taxonomic analysis, while we employed double-digest restriction site-associated DNA sequencing (ddRAD-seq) for population genomics studies. Complementary shallow genomic sequencing of representative accessions enabled chloroplast phylogeny reconstruction. By integrating these multidimensional datasets, we aimed to address the persistent challenges: 1) disentangling phylogenetic relationships amid morphological convergence in ser. *P*, ser. *B*, and related taxa; 2) establishing biologically meaningful species boundaries; and 3) finding potential gene flow and hybridization events. This multidisciplinary framework advances integrative taxonomy in *Cotoneaster* by reconciling classical morphological criteria with genomic evidence, ultimately providing a modest reference for refining the genus’ taxonomic architecture.

## 2 Materials and methods

### 2.1 Studied species and sample collection

**Figure 2:**
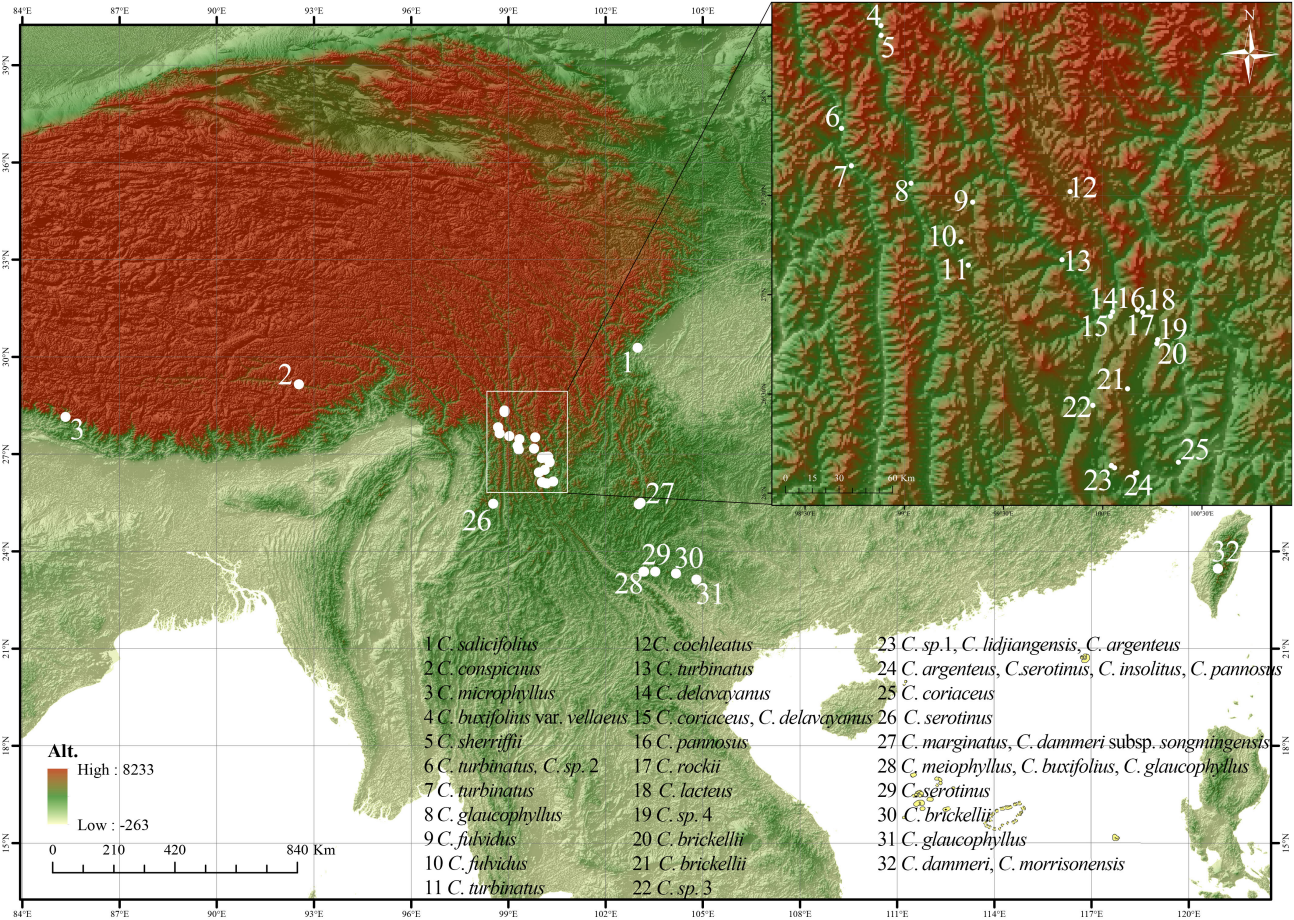
Collection map of the *Cotoneaster buxifolius* complex and related taxa, with location coded from 1 to 32.

Building upon previous phylogenetic frameworks (Meng et al., 2021; Fryer and Hylmö, 2009), this research sampled 29 taxa including four morphologically ambiguous entities (**Table 1**). The sampling strategy targeted nine species from ser. *P*, six from ser. *B*, and nine additional taxa from phylogenetically adjacent series *Salicifolii* T. T. Yu (*S*), *Conspicui* G. Klotz (*C*), *Radicantes* G. Klotz (*R*), and *Microphylli* T. T. Yu (*M*). Field collections spanned 43 natural populations (458 accessions; 2–23 individuals per population) across major distribution areas: Sichuan, Yunnan, Tibet (China), Taiwan (China), and Rasuwa District (Nepal) (**Figure 2**). Georeferencing was performed using the Ovital Map application, with spatial metadata detailed in **Supplementary Table S1**. Voucher specimens were deposited at Sun Yat-sen University Herbarium (SYS). Fresh leaves were desiccated in silica gel for subsequent genomic analyses.

**Table 1:** Taxonomic status and classification of studied *Cotoneaster buxifolius* complex and related taxa.

Fryer and Hylmö (2009)		Treatment	
Species	Ser.	Catalogue of Life China (2024)	WFO Plant List: World Flora Online (2024)
*C. brickellii*	P	Accepted	Accepted
*C. coriaceus*	P	Accepted	Accepted
*C. lacteus*	P	*C. coriaceus*	*C. coriaceus*
*C. pannosus*	P	Accepted	Accepted
*C. turbinatus*	P	Accepted	Accepted
*C. fulvidus*	P	*C. hebephyllus* var. *fulvidus*	*C. hebephyllus* var. *fulvidus*
*C. glaucophyllus*	P	Accepted	Accepted
*C. serotinus*	P	*C. glaucophyllus* var. *serotinus*	*C. glaucophyllus* var. *serotinus*
*C. meiophyllus*	P	*C. glaucophyllus* var. *meiophyllus*	*C. glaucophyllus* var. *meiophyllus*
*C. buxifolius*	B	Accepted	Accepted
*C. argenteus*	B	*C. buxifolius* var. *buxifolius*	*C. buxifolius*
*C. lidjiangensis*	B	*C. buxifolius* var. *buxifolius*	*C. pannosus* var. *pannosus*
*C. rockii*	B	*C. buxifolius* var. *rockii*	Accepted
*C. insolitus*	B	*C. buxifolius* var. *rockii*	*C. integrifolius*
*C. buxifolius* var. *vellaeus* (Unrecorded)	B	Accepted	*C. microphyllus*
*C. delavayanus*	B	Accepted	Accepted
*C. cochleatus*	R	*C. microphyllus* var. *cochleatus*	Accepted
*C. dammeri*	R	Accepted	Accepted
*C. dammeri* subsp. *songmingensis*	R	*C. dammeri* var. *dammeri*	*C. dammeri*
*C. morrisonensis*	R	Accepted	Accepted
*C. conspicuus* Comber ex Marquand	C	*C. conspicuus* (Messel) Messel	Accepted
*C. sherriffii*	C	Accepted	Accepted
*C. marginatus*	M	*C. buxifolius* var. *marginatus*	*C*. *integrifolius*
*C. microphyllus*	M	Accepted	Accepted
*C. salicifolius*	S	Accepted	Accepted
*C*. sp. 1	B	/	/
*C*. sp. 2	S	/	/
*C*. sp. 3	M	/	/
*C*. sp. 4	P	/	/

S, Ser. *Salicifolii* < Sect. *Densiflori*; P, Ser. *Pannosi* < Sect. *Densiflori*; B, Ser. *Buxifolii* < Sect. *Alpigeni*; M, Ser. *Microphylli* < Sect. *Alpigeni*; R, Ser. *Radicantes* < Sect. *Alpigeni*; C, Ser. *Conspicui* < Sect. *Alpigeni*.

### 2.2 Morphological characterization and analysis

Detailed morphological observation and analysis incorporated 1–20 specimens per population. Morphological characteristics of the *Cotoneaster brickellii* J. Fryer and B. Hylmö WS and *Cotoneaster meiophyllus* (W. W. Sm.) G. Klotz LHS populations were recorded based on field observations and literature descriptions (Fryer and Hylmö, 2009). Six quantitative and 16 qualitative traits were identified for analysis with qualitative traits encoded as integers (**Supplementary Table S2**). Principal component analysis (PCA) implemented in FactoMineR v2.9 (Lê et al., 2008) utilized all 22 traits (**Supplementary Table S3**), visualized using biplots showing species distributions in PCA space and trait contributions. Gower’s distance matrix was calculated using gower v0.1.2 (Gower, 1971), based on the mean of four quantitative traits (leaf length, leaf width, leaf length-to-width ratio, and petiole length) and 16 coded qualitative traits (**Supplementary Table S4**). Hierarchical clustering was performed using the average method by SciPy v. 1.11.2 (Virtanen et al., 2020), and a dendrogram was generated using Matplotlib v. 3.7.2 (Hunter, 2007) to illustrate population relationships.

### 2.3 Plastid assembly and phylogenetic tree construction

Shallow genome sequencing of one to two– randomly selected individuals per population was conducted on the Illumina NovaSeq platform (150-bp paired-end reads; JieRui BioScience, Guangzhou, China). Chloroplast genome assembly followed a four-stage pipeline: quality trimming with fastp v0.23.4 (Chen et al., 2018), *de novo* assembly via GetOrganelle v1.7.7.0 (Jin et al., 2020) using *C. lacteus* (MK605517) as reference, iterative polishing with Pilon v1.24 (Walker et al., 2014), and collinearity verification using MUMmer v4.0.0rc1 (Marçais et al., 2018). The final plastid genome dataset combined 29 newly assembled plastid genomes in this study with 64 publicly available plastid genomes of *Cotoneaster* species and *Eriobotrya deflexa* (Hemsl.) Nakai downloaded from the National Center for Biotechnology Information (NCBI) nucleotide database (https://www.ncbi.nlm.nih.gov). The corresponding accession numbers are listed in **Supplementary Table S5**. Multiple sequence alignment using MAFFT v. 7.505 (Katoh and Standley, 2013) with default settings was refined with Gblocks v0.91b (Castresana, 2000) to remove half-gap regions. IQ-TREE multicore v1.6.12 (Nguyen et al., 2015) was utilized to construct maximum likelihood phylogenetic trees. The Shimodaira–Hasegawa approximate likelihood ratio test (SH-aLRT) and ultrafast bootstrap (UFboot) values were both set to 10,000 to evaluate the reliability of the phylogenetic tree topology and node support.

### 2.4 RAD sequencing and data processing

Genomic DNA was extracted from silica-dried leaf samples using a modified cetyl-trimethylammonium bromide (CTAB) method as described by Doyle and Doyle (1987) and Yang et al. (2010). Leaf tissues, excluding veins, were flash-frozen in liquid nitrogen for 10 min, homogenized with magnetic beads using a tissue homogenizer, and lysed in 1.5 mL STE buffer (10-min inversion mixing). After centrifugation (5,000 ×*g*, 5 min), the pellet was resuspended in preheated 3% CTAB buffer (65°C, 45 min, gentle inversion every 10 min). Subsequent steps included chloroform:isoamyl alcohol (24:1) extraction (twice), RNase A treatment (37°C, 30 min), isopropanol precipitation (−20°C, 1 night), and three washes with 70% ethanol. DNA pellets were air-dried and dissolved in 100 μL TE buffer. All centrifugations were performed at 12,000 ×*g* for 10 min using a pre-cooled centrifuge (4°C). ddRAD libraries were prepared and sequenced by JieRui BioScience Co. Ltd. (Guangzhou, China) on an Illumina NovaSeq platform with 150-bp paired-end reads. A total of 458 individuals from 43 populations were included in the population genomic analysis. The raw ddRAD-seq data for all *Cotoneaster* taxa generated for this study was uploaded to GenBank under the BioProject PRJNA1196588.

Raw data quality was evaluated using FastQC v0.12.1 (Andrews, 2010) and MultiQC v1.15 (Ewels et al., 2016), followed by adapter and low-quality base trimming using fastp v0.23.4 (Chen et al., 2018) and cutadapt v4.4 (Martin, 2011). Filtered reads were aligned to the genome of *C. glaucophyllus* (Meng et al., 2024) using the BWA-MEM algorithm with parameters -B 3 and -O 5,5, as implemented in BWA v0.7.17-r1188 (Li, 2013). Variant calling and refinement were performed using an established GATK v4.3.0.0 (McKenna et al., 2010) workflow. Read group information was first incorporated into alignment files using the AddOrReplaceReadGroups module. Per-sample GVCF files were subsequently generated using HaplotypeCaller, followed by the assembly of a consolidated GenomicsDB using GenomicsDBImport for chromosome-specific variant extraction. Merged VCF files underwent rigorous quality filtration via VariantFiltration, retaining only single-nucleotide polymorphisms (SNPs) satisfying the following thresholds: quality by depth (QD) > 2.0, Phred-scaled quality score (QUAL) > 30.0, Fisher’s strand bias (FS) < 60.0, mapping quality (MQ) > 40.0, mapping quality rank sum (MQRankSum) > −12.5, and read position rank sum (ReadPosRankSum) > −8.0. The filtered variant set was further processed using bcftools v1.17 (Danecek et al., 2021) to retain strictly biallelic sites exhibiting ≤ 15% missing data, a minimum minor allele count (MAC) of 3, and minor allele frequency (MAF) ≥ 5%. Fourfold degenerate (4D) sites were systematically identified through iTools v0.25 (He et al., 2013) using coordinate annotations from the *C. glaucophyllus* reference genome (Meng et al., 2024). Linkage disequilibrium (LD) pruning was implemented in PLINK v1.90b6.26 (Purcell et al., 2007) using a sliding window approach (50-SNP windows advanced in 10-SNP increments), with sites demonstrating pairwise LD (r^2^) > 0.1 excluded from downstream analyses.

### 2.5 Phylogenetic analyses and population genetics based on RAD-seq

The maximum likelihood phylogenetic tree was reconstructed using IQ-TREE multicore v1.6.12 (Nguyen et al., 2015) based on 4D sites derived from the RAD-seq dataset, with SH-aLRT and UFboot values set to 5,000 and 10,000, respectively. Population structure inference was implemented through ADMIXTURE v1.3.0 (Alexander et al., 2009) using a curated dataset of non-missing, linkage-pruned 4D sites, testing *K* = 1–40 with 10 replicates per *K*. Cross-validation error minimization determined optimal genetic clusters, visualized using pophelper v2.3.1 (Francis, 2017). Principal component analysis (PLINK v1.90b6.26) of SNPs with 85% completeness generated covariance matrices, with Python-customized scripts visualizing dispersion patterns along the first two principal components.

## 3 Results

### 3.1 Morphological analyses

**Figure 3:**
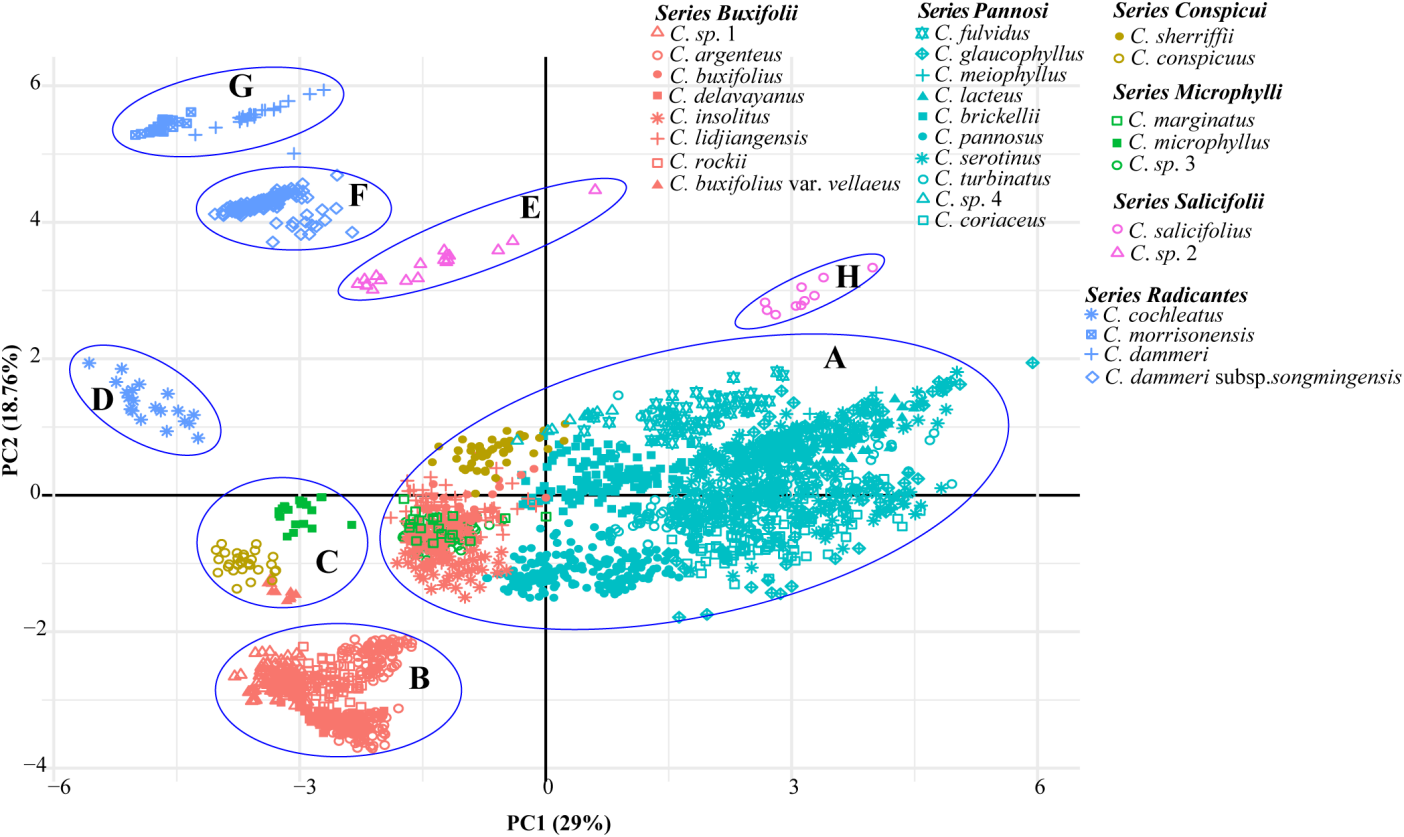
Principal component analysis (PCA) of 43 populations of *Cotoneaster buxifolius* complex and related taxa based on 22 morphological traits. Six colors denote taxonomic series, with geometric markers differentiating taxa. Clusters are labeled with **(A–H)** and circles.

**Figure 4:**
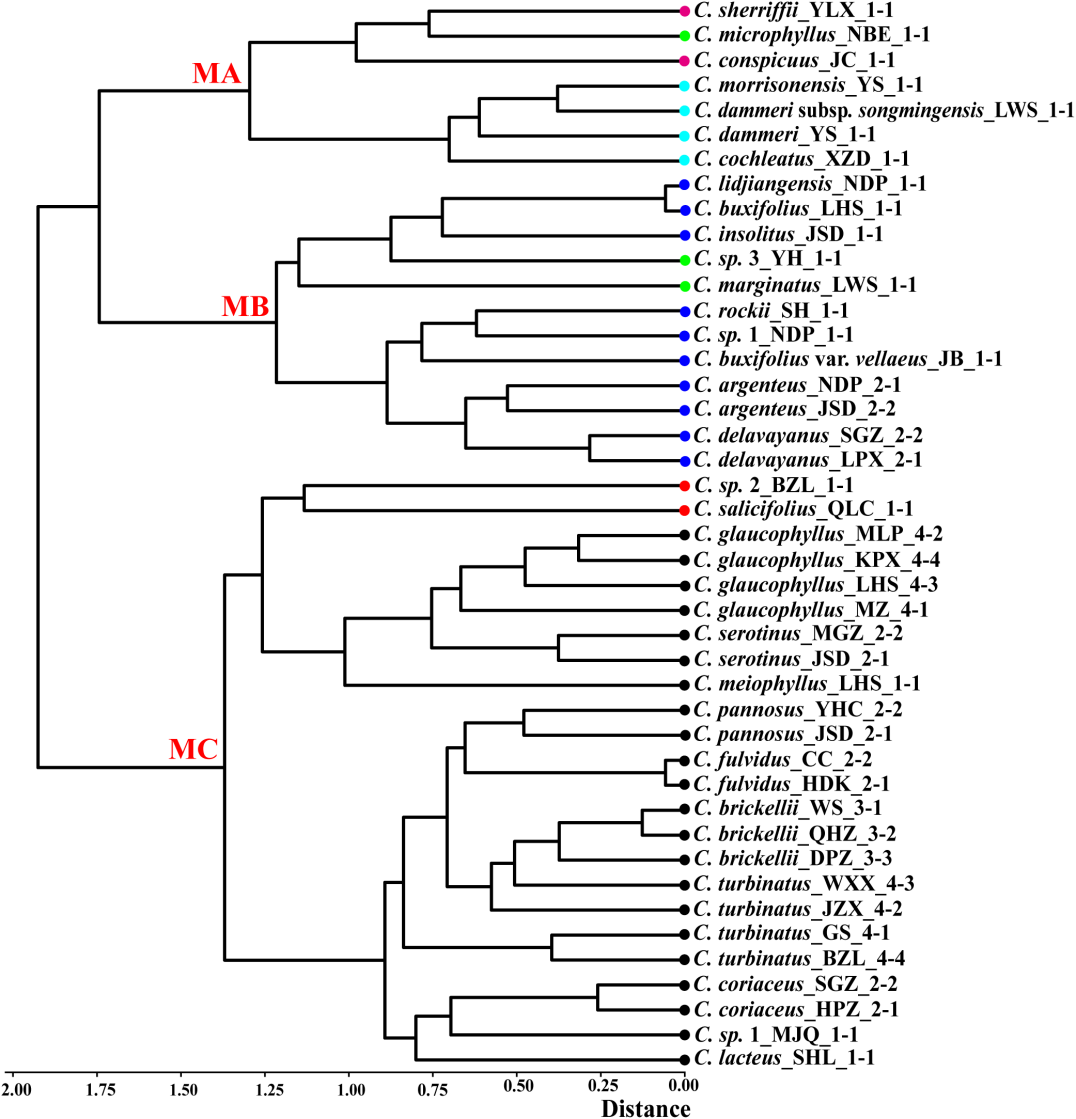
Hierarchical clustering of 43 populations from *Cotoneaster buxifolius* complex and related taxa based on 20 morphological traits, with major clades labeled MA (morphological group A), MB (morphological group B), and MC (morphological group C). Color dot codes: red = ser. *Salicifolii*, pink = ser. *Conspicui*, green = ser. *Microphylli*, mint blue = ser. *Radicantes*, blue = ser. *Buxifolii*, and black = ser. *Pannosi*.

PCA was performed on 29 taxa using 22 morphological traits. The first two principal components explained 29% and 18.76% of the total variance. Leaf length, leaf width, rooting habit, and fertile shoot composition significantly contributed to the variance of PC1 and PC2, while traits such as upper leaf surface color showed minimal influence (**Supplementary Figure S2**). The PCA plot elucidated the relationships among different series (**Figure 3**). All species in ser. *P*, together with *C. buxifolius*, *Cotoneaster lidjiangensis* G. Klotz and *Cotoneaster insolitus* G. Klotz (ser. *B*), *Cotoneaster sherriffii* G. Klotz (ser. *C*), and *C. marginatus* (ser. *M*), formed a compact and distinct group A. Four additional ser. *B* species formed Group B, while *C. buxifolius* var. *vellaeus* (Franch.) G. Klotz (ser. *B*) clustered with *C. conspicuus* (ser. *C*) and *Cotoneaster microphyllus* Wall. ex Lindl. (ser. *M*) in Group C. The four ser. *R taxa*, split into groups D (*C. morrisonensis* and *C. dammeri* C. K. Schneider), F (*C. dammeri* subsp. *songmingensis*), and G [*Cotoneaster cochleatus* (Franchet) G. Klotz], while the two species in ser. *S* formed groups E (*C*. sp. 2) and H (*Cotoneaster salicifolius* Franch.). Hierarchical clustering divided all taxa into three major clades (**Figure 4**). Clade MC comprised taxa from ser. *P* and ser. *S*, further divided into two subgroups: one containing *Cotoneaster serotinus* Hutch., *C. glaucophyllus*, and *C. meiophyllus* clustered with ser. *S* taxa, while the other encompassed the remaining members of ser. *P*. Clade MB integrated taxa from ser. *B* alongside *C.* sp. 3 and *C. marginatus* (both morphologically assigned to ser. *M*). Within this clade, *C. lidjiangensis*, *C. buxifolius*, and *C. insolitus* clustered with *C.* sp. 3 and *C. marginatus*, forming one subclade, while the remaining *series B* taxa—*Cotoneaster rockii* G. Klotz, *C.* sp. 1, *C. buxifolius* var. *vellaeus*, *Cotoneaster argenteus* G. Klotz, and *Cotoneaster delavayanus* G. Klotz—grouped into another distinct subclade. Clade MA consisted of two lineages: an independent branch representing ser. *R* and the other distinct cluster formed by ser. *C* taxa and *C. microphyllus* (classified under ser. *M*).

### 3.2 Plastid phylogeny

Shallow sequencing produced over 6 Gb per sample, with Guanine-Cytosine (GC) content ranging from 38.42% to 42.47% and Q20 scores surpassing 96%. *De novo* assembly generated 29 complete circular chloroplast genomes, spanning 159,152 to 159,841 bp in length. Alignment of both NCBI-downloaded and newly assembled chloroplast genomes followed by half-gap region trimming yielded a consensus sequence of 159,394 bp. Using ModelFinder (Kalyaanamoorthy et al., 2017) under the Bayesian information criterion (BIC), the TVM+F+R3 model was identified as the optimal nucleotide substitution model for chloroplast genome phylogenetic reconstruction.

**Figure 5:**
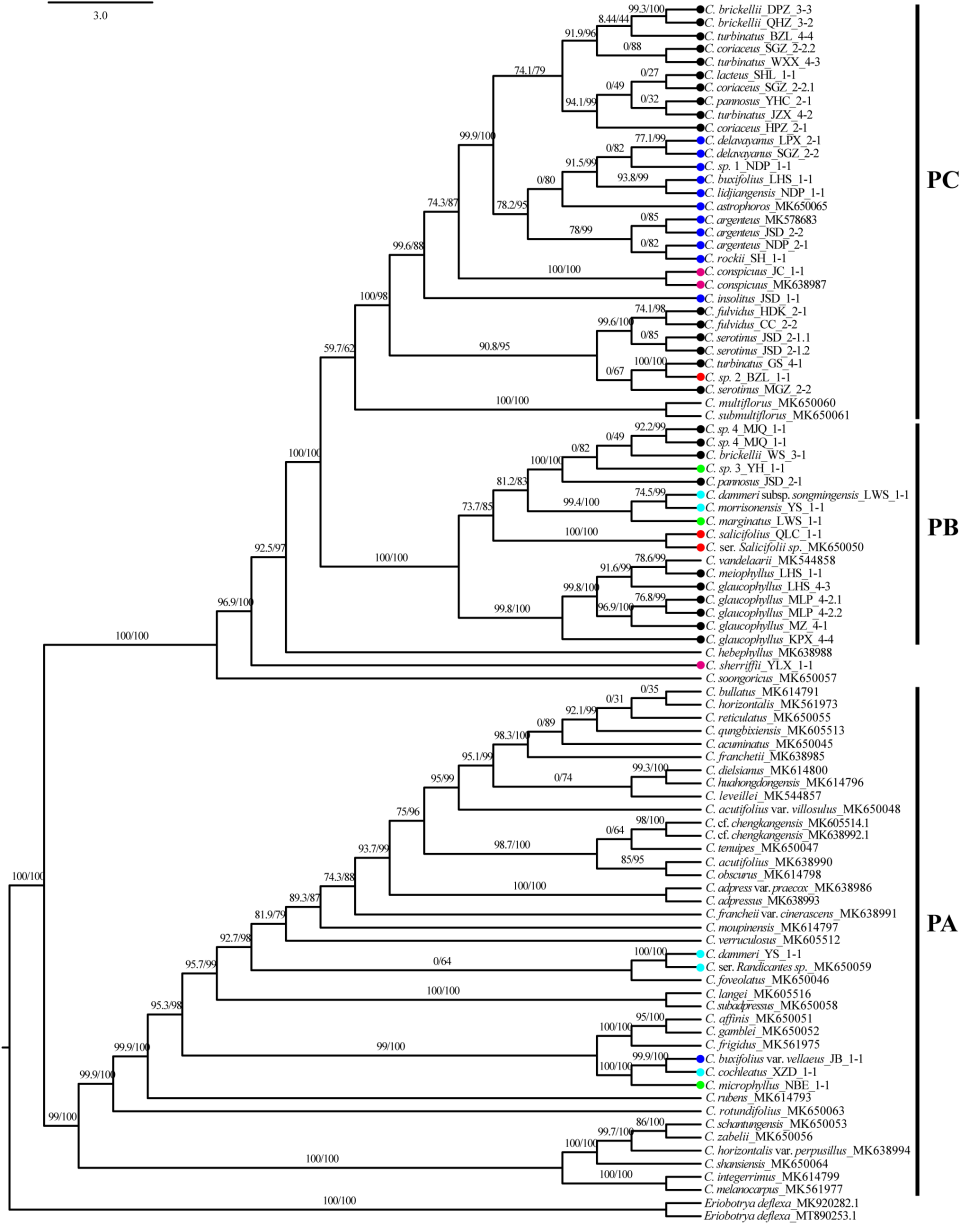
Chloroplast phylogenetic tree, SH-aLRT, and UFboot values shown above the branches, with main clades marked as PA (plastid clade A), PB (plastid clade B), and PC (plastid clade C). Color dot codes: red = ser. *Salicifolii*, pink = ser. *Conspicui*, green = ser. *Microphylli*, mint blue = ser. *Radicantes*, blue = ser. *Buxifolii*, and black = ser. *Pannosi*. SH-aLRT, Shimodaira–Hasegawa approximate likelihood ratio test; UFboot, ultrafast bootstrap.

Phylogenetic reconstruction using 93 plastid sequences resolved three primary clades: PA, PB, and PC (**Figure 5**). Clade PA was predominantly composed of taxa from subgenus *Cotoneaster*. Within this clade, *C. buxifolius* var. *vellaeus*, *C. cochleatus*, and *C. microphyllus* formed a fully supported lineage (SH-aLRT/UFboot: 100/100), while *C. dammeri* was embedded within PA as an independent, maximally supported branch (SH-aLRT/UFboot: 100/100). Clade PB comprised two subclades with strong or moderate phylogenetic support. The first subclade grouped *C. glaucophyllus* and *C. meiophyllus* (SH-aLRT/UFboot: 99.8/100). The second subclade contained three lineages: a fully supported monophyletic lineage of *Cotoneaster pannosus* Franch. (JSD population), *C. brickellii* (WS population), *C.* sp. 4, and *C.* sp. 3 (ser. *M*; SH-aLRT/UFboot: 100/100); a distinct lineage comprising *C. dammeri* subsp. *songmingensis*, *C. morrisonensis*, and *C. marginatus* (SH-aLRT/UFboot: 99.4/100); and an independent branch formed by *C. salicifolius* (SH-aLRT/UFboot: 100/100). Clade PC exhibited polyphyletic structuring of ser. *P* intermingled with other series. At its base, a moderately supported lineage (SH-aLRT/UFboot: 90.8/95) included *Cotoneaster fulvidus* (W. W. Sm.) G. Klotz, *C. serotinus*, *Cotoneaster turbinatus* Craib (GS population), and *C.* sp. 2. The majority of ser. *P* taxa formed a monophyletic group (SH-aLRT/UFboot: 99.9/100) sister to the primary ser. *B* lineage, which itself was monophyletic except for the exclusion of *C. insolitus* and *C. buxifolius* var. *vellaeus*. *C. conspicuus* was resolved as basal to clade PC. Taxa from ser. *C*, *R*, *M*, and *S* showed no monophyletic clustering across the phylogeny.

### 3.3 Phylogenetic tree based on RAD-seq

Initial variant calling through GATK identified 9,855,426 raw SNPs. These variants underwent rigorous filtering (minor allele count ≥ 3, minor allele frequency ≥ 5%, and genotype completeness ≥ 85%), resulting in 534,022 high-confi dence SNPs. From this filtered dataset, 89,769 4D sites were extracted, and LD pruning further refined the dataset to 100% complete 40,569 unlinked 4D sites.

**Figure 6:**
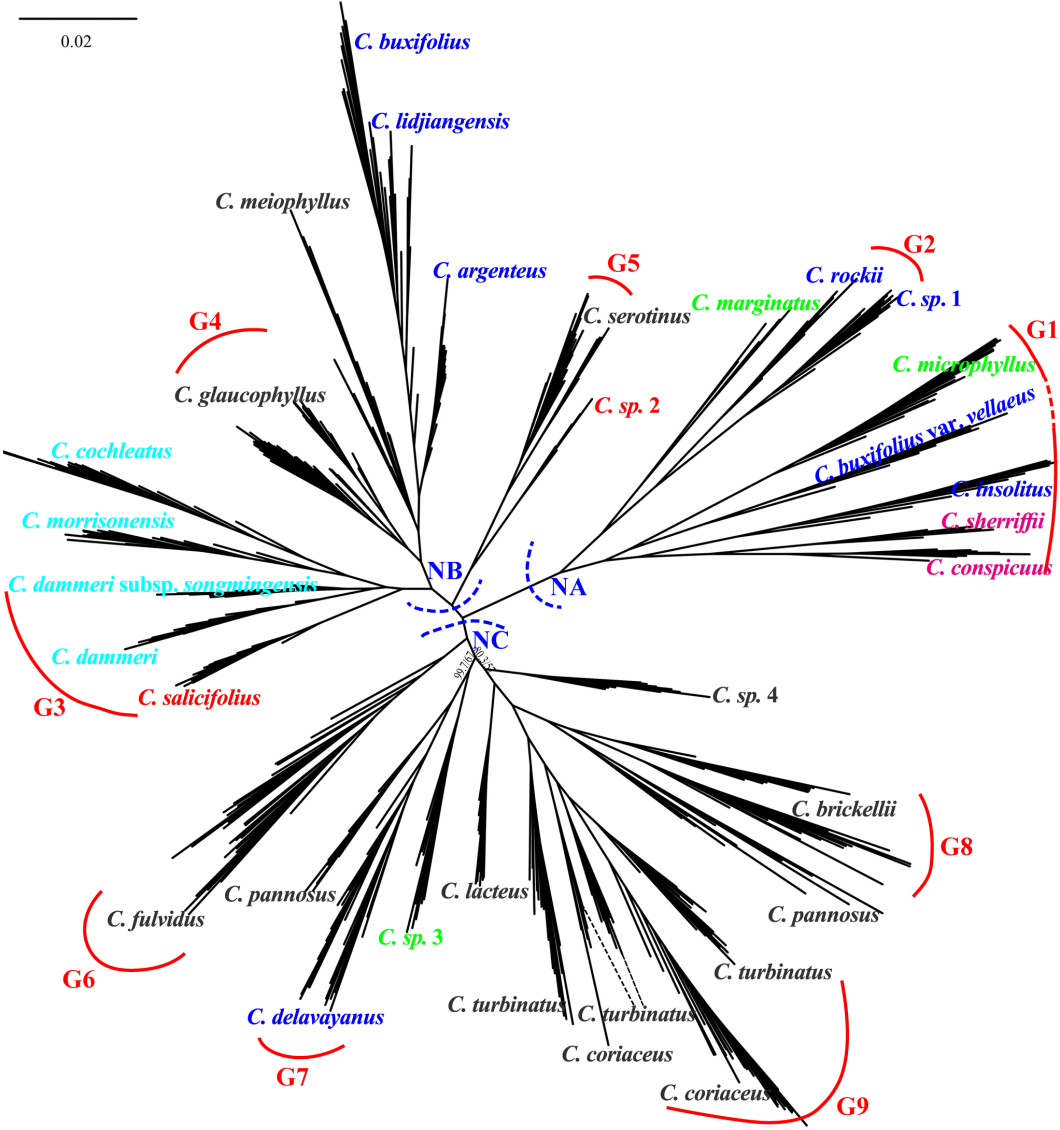
Unrooted phylogenetic tree constructed using 4D sites, with major clades labeled NA (nuclear clade A), NB (nuclear clade B), and NC (nuclear clade C). Main branches are supported by SH-aLRT ≥ 80% and UFboot ≥ 95% unless otherwise indicated. G1 to G9 represent species with independent gene pools at admixture analysis *K* = 9. Color taxon name codes: red = ser. *Salicifolii*, pink = ser. *Conspicui*, green = ser. *Microphylli*, mint blue = ser. *Radicantes*, blue = ser. *Buxifolii*, and black = ser. *Pannosi*. 4D, fourfold degenerate; SH-aLRT, Shimodaira–Hasegawa approximate likelihood ratio test; UFboot, ultrafast bootstrap.

Maximum likelihood phylogenetic analysis based on the 89,769 4D sites resolved three primary clades (NA, NB, and NC) with robust support, as evidenced by high SH-aLRT and UFboot values for most major clades with SH-aLRT ≥ 80% and UFboot ≥ 95% (**Figure 6**). Individuals from the same population predominantly formed monophyletic clusters. Clade NA comprised two taxa of ser. *C*, four taxa from ser. *B* (*C. rockii*, *C*. sp. 1, *C. buxifolius* var. *vellaeus*, and *C. insolitus*), and two taxa from ser. *M* (*C. marginatus* and *C. microphyllus*). Clade NB consisted of three ser. *B* taxa (*C. buxifolius*, *C. lidjiangensis*, and *C. argenteus*), three ser. *P* taxa (*C. glaucophyllus*, *C. meiophyllus*, and *C. serotinus*), four taxa of ser. *R*, and *C. salicifolius* from ser. *S*. Clade NC encompassed predominantly ser. *P* taxa (*C.* sp. 4, *C. brickellii*, *C. pannosus*, *C. turbinatus*, *C. coriaceus*, *C. lacteus*, and *C. fulvidus*), along with *C. delavayanus* from ser. *B* and *C.* sp. 3 from ser. *M*. Notably, ser. *M*, ser. *B*, and ser. *P* exhibited polyphyletic distributions across the phylogeny. Additionally, the two *C. pannosus* populations failed to form a monophyletic group despite the high overall support for major clades.

### 3.4 Population genomic analysis

**Figure 7:**
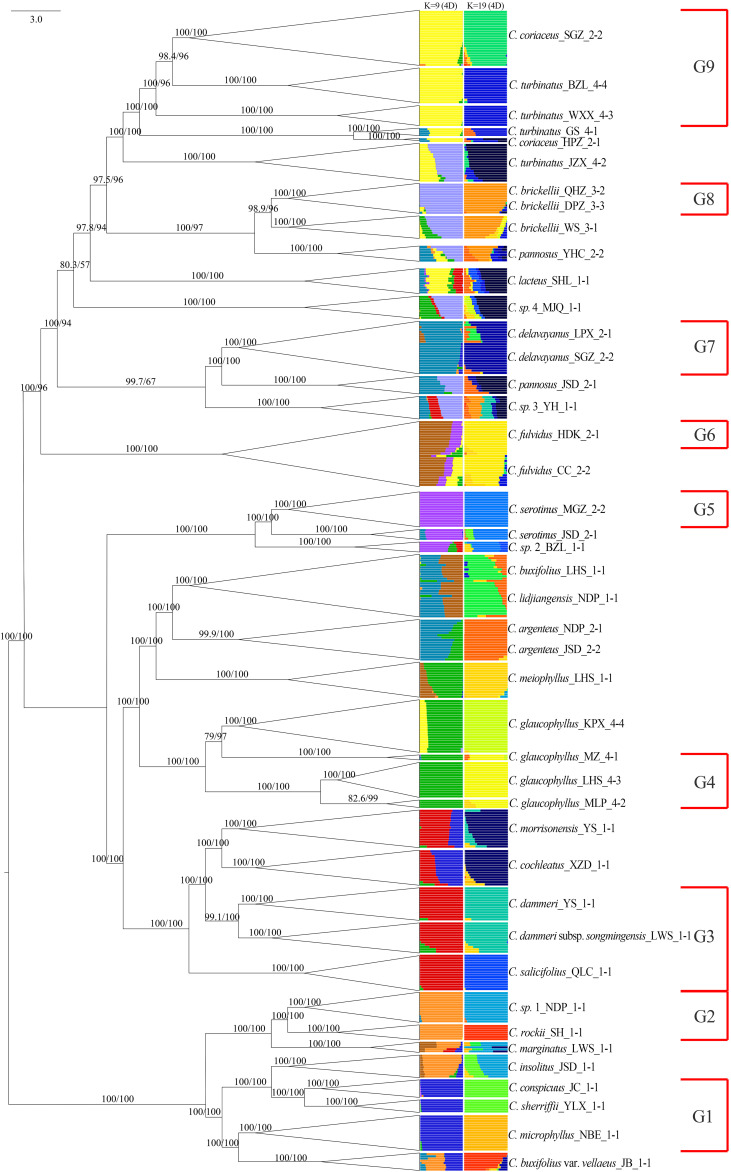
Admixture results (*K* = 9 and 19) using unlinked 4D sites combined with the phylogenetic tree constructed. G1 to G9 represent species with independent gene pools at admixture analysis *K* = 9. 4D, fourfold degenerate.

Population genetic structure analysis using the Admixture software revealed dynamic clustering patterns across *K* values. The cross-validation error stabilized at *K* = 9 and reached its minimum at *K* = 19 (**Supplementary Figure S3**). At *K* = 9, nine distinct genetic clusters were identified: 14 species exhibited near-exclusive ancestry (> 95% assignment to single clusters), except for *C. fulvidus* (> 70% cluster purity), while the remaining taxa displayed admixture from two or more gene pools (**Figure 7**). Mapping these clusters onto the nuclear phylogeny demonstrated spatial congruence: gene pools G1 and G2 localized predominantly within clade NA; G3, G4, and G5 were primarily located in clade NB; and G6 through G9 were distributed within clade NC. At *K* = 19, five species (*C. marginatus*, *C. insolitus*, *C. pannosus*, *C.* sp. 1, and *C. lacteus*) still maintained genetic admixture of two or more gene pools, while five pairs of taxa shared identical cluster assignments: *C. conspicuus* and *C. sherriffii*, *C. dammeri* subsp. *songmingensis* and *C. dammeri*, *C. morrisonensis* and *C. cochleatus*, *C. buxifolius* and *C. lidjiangensis*, and *C. serotinus* and *C.* sp. 2. Furthermore, seven species, *C. microphyllus*, *C. rockii*, *C.* sp. 1, *C. salicifolius*, *C. meiophyllus*, *C. argenteus*, and *C. coriaceus*, formed a novel gene pool separately.

**Figure 8:**
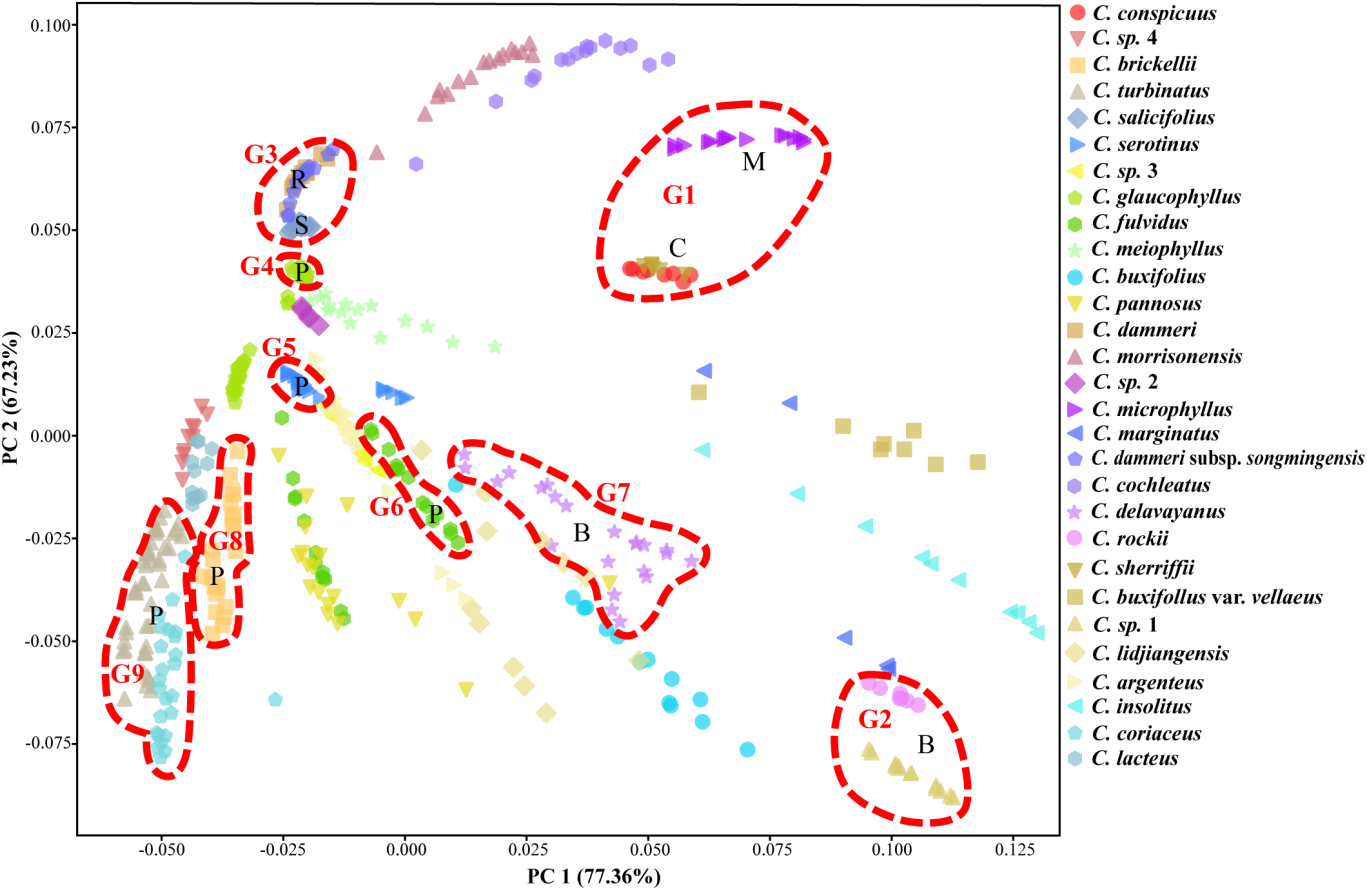
Principal component analysis (PCA) of 43 populations from *Cotoneaster buxifolius* complex and related taxa based on 85% completeness SNPs, with nine gene pools, G1 to G9, marked by circles. SNPs, single-nucleotide polymorphisms.

The PCA of 458 individuals (**Figure 8**) showed partial species differentiation, such as *C. microphyllus*, *C. rockii*, and *C.* sp. 1, although a clear and distinct pattern was not fully established. The distribution of the 14 species representing gene pools G1 to G9 exhibited structured spatial relationships: G8 and G9 demonstrated close proximity, G3 and G4 formed a tightly clustered group, G5–G7 occupied central positions, and G1 and G2 were peripherally distributed. Notably, certain taxa exhibited displayed near-identical genetic profiles in PCA space, as exemplified by *C. conspicuus*–*C. sherriffii* and *C. dammeri*–*C. dammeri* subsp. *songmingensis*.

### 3.5 Cytonuclear conflict in phylogenetic trees

**Figure 9:**
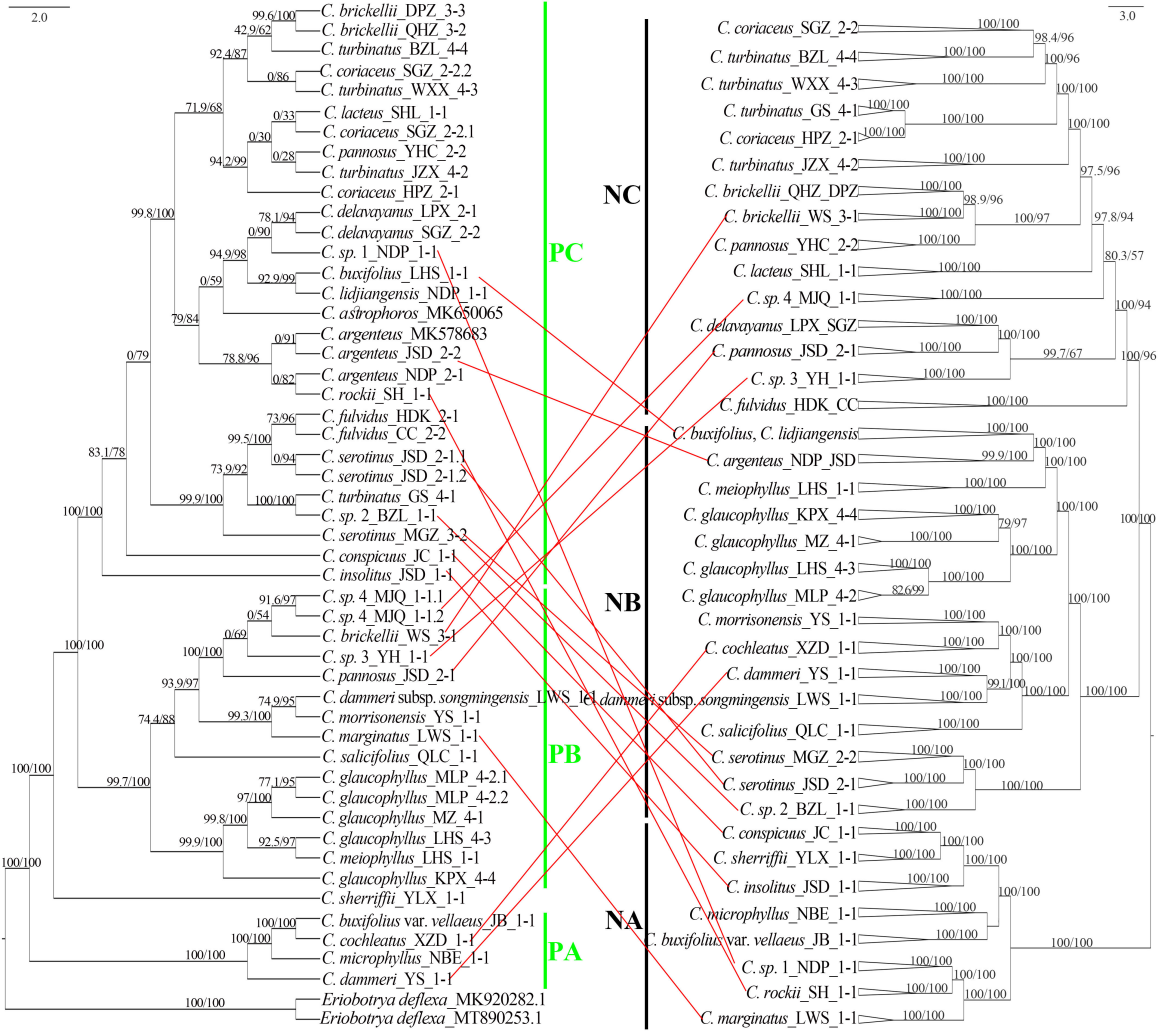
Chloroplast genome tree (left: plastid clades PA–PC) and 4D site-based phylogenetic tree (right: nuclear clades NA–NC), with SH-aLRT and UFboot values marked above the branches. Red lines highlight nuclear–plastid discordance (topological conflicts between plastid and nuclear trees). 4D, fourfold degenerate; SH-aLRT, Shimodaira–Hasegawa approximate likelihood ratio test; UFboot, ultrafast bootstrap.

Comparative analysis of plastid and nuclear phylogenies revealed cytonuclear discordance in 18 populations spanning 16 species (**Figure 9**). Among the eight populations in the nuclear clade NA, *C. marginatus* was assigned to the plastid clade PB, while *C. rockii*, *C.* sp. 1, *C. insolitus*, and *C. conspicuus* grouped within the plastid clade PC. Of the 17 populations in nuclear clade NB, *C. cochleatus* and *C. dammeri* were placed in the plastid clade PA, whereas seven populations of *C. serotinus*, *C.* sp. 2, *C. argenteus*, *C. buxifolius*, and *C. lidjiangensis* resided in the plastid clade PC. In the nuclear clade NC (18 populations), *C. brickellii* (WS), *C. pannosus* (JSD), *C.* sp. 3, and *C.* sp. 4 clustered in the plastid clade PB. Furthermore, three of the 14 species representing nine gene pools, *C. dammeri*, *C. conspicuus*, and *C.* sp. 1, maintained cytonuclear discordance even after the exclusion of putative hybrids (**Figure 9**).

### 3.6 Phylogenetic tree reconstruction after removing putative hybrids

**Figure 10:**
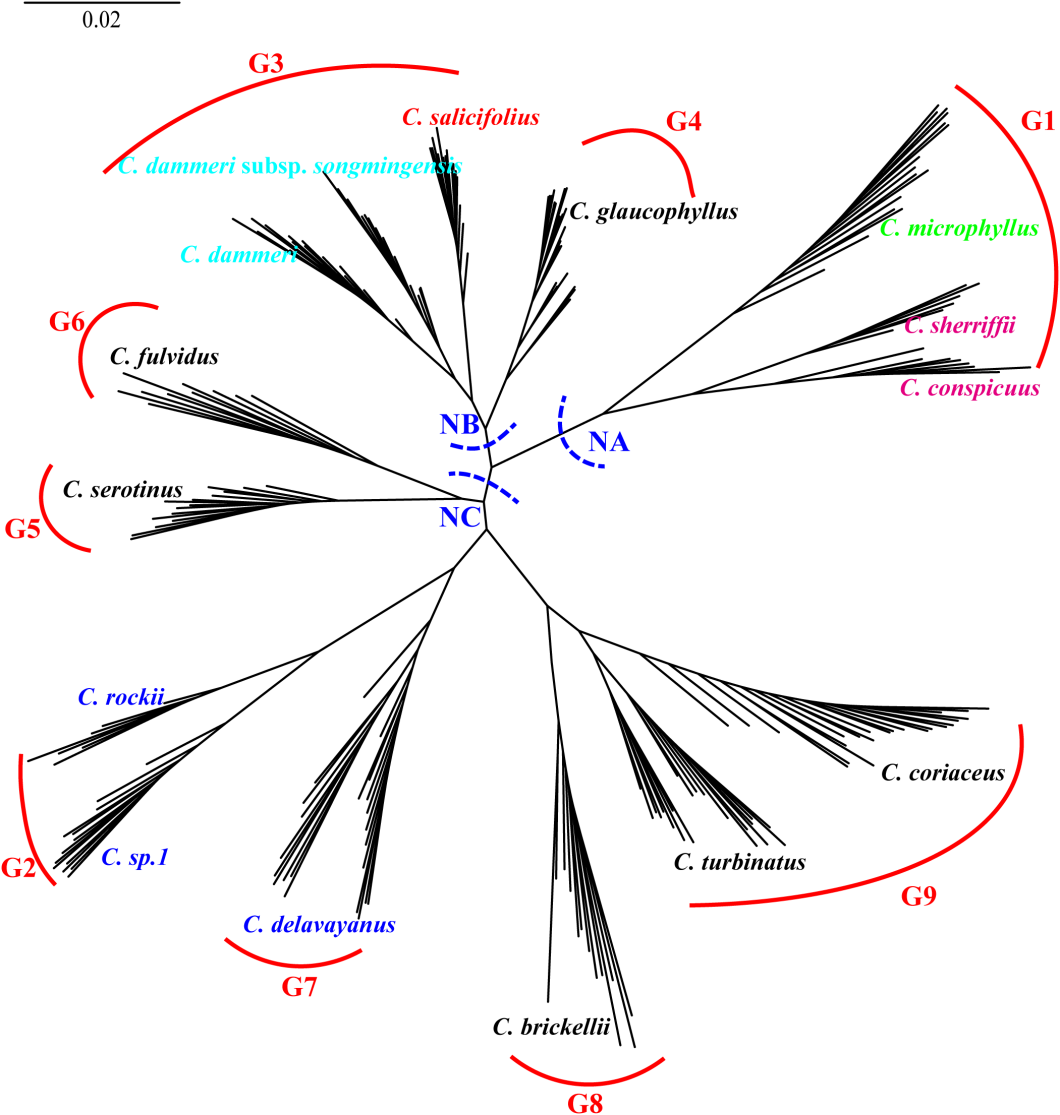
4D site-based phylogenetic tree after removing putative hybrids, with major clades labeled NA (nuclear clade **A**), NB (nuclear clade **B**), and NC (nuclear clade **C**). Main branches are supported by SH-aLRT ≥ 80% and UFboot ≥ 95% unless otherwise indicated. G1 to G9 represent species with independent gene pools at admixture analysis *K* = 9. 4D, fourfold degenerate; SH-aLRT, Shimodaira–Hasegawa approximate likelihood ratio test; UFboot, ultrafast bootstrap.

Integrating admixture results (*K* = 9) with nuclear phylogenetic relationships, we reconstructed a maximum likelihood phylogeny for 20 genetically non-admixed populations representing nine gene pools (**Figure 10**). In this tree, clade NA encompassed three species representing gene pool G1: *C. conspicuus* and *C. sherriffii* (ser. *C*), and *C. microphyllus* (ser. *M*; **Figure 10**). Clade NB united *C. salicifolius* (ser. *S*; G3), *C. glaucophyllus* (ser. *P*; G4), *C. dammeri*, and *C. dammeri* subsp. *songmingensis* (ser. *R*; G4). Clade NC exhibited a complex hierarchical structure, incorporating five ser. *P* and three ser. *B* taxa. Gene pools G8 (*C. brickellii*) and G9 (*C. turbinatus* and *C. coriaceus*) formed a monophyletic group that subsequently diverged from the sister gene pools G2 (*C. rockii* and *C.* sp. 1) and G7 (*C. delavayanus*) and later clustered with the monophyletic group comprising G5 (*C. serotinus*) and G6 (*C. fulvidus*). All terminal clades corresponding to single taxonomic units received strong nodal support (SH-aLRT ≥ 80% and UFboot ≥ 95%).

## 4 Discussion

The resolution of interspecific relationships within *Cotoneaster* is confounded by intersecting evolutionary mechanisms including hybridization, incomplete lineage sorting, and polyploidization. Current multi-dataset analyses revealed persistent topological conflicts among phylogenies reconstructed from morphological traits, chloroplast genomes, and nuclear RAD-seq data. Notably, conspecific populations frequently failed to form monophyletic clusters across these reconstructions, a pattern compounded by admixture analyses demonstrating pervasive gene pool mixing among nominal species. These observations collectively underscore the prevalence of reticulate evolutionary processes and gene flow-mediated phylogenetic discordance in the genus. The recurrent cytonuclear discordances, non-monophyly of morphologically defined taxa, and extensive interspecific admixture necessitate a framework integrating phylogenomic networks and demographic modeling to disentangle historical hybridization signals from incomplete lineage sorting dynamics.

### 4.1 Conflicts between morphological and genetic data: implications for integrative taxonomy

The genus *Cotoneaster* presents formidable challenges in species delimitation due to extensive morphological plasticity arising from polyploidy, recurrent hybridization, and adaptive divergence, compounded by apomixis that generates morphologically intermediate forms (Rothleutner et al., 2016; Fryer and Hylmö, 2009). Sympatric populations exhibit heightened hybridization frequencies, amplifying morphological ambiguity and taxonomic complexity (Li et al., 2014; Li et al., 2017).

PCA based on 22 morphological traits revealed that species in ser. *M*, *S*, *R*, and *C* could be effectively distinguished, whereas most species in ser. *B* aggregated into two groups, apart from *C. buxifolius* var. *vellaeus* (**Figure 3**). In contrast, ser. *P* species showed pronounced morphological overlap (**Figure 3**), likely reflecting subtle morphological divergence within the *C. buxifolius* complex where leaf dimensions and lateral vein depression gradients create phenotypic continuity. Transitional forms bridging *C. buxifolius*, *C. pannosus*, and *C. brickellii* exemplify this continuum. Although branchlet arrangement differentiates ser. *B* (predominantly spiral) from ser. *P* (spiral/distichous), partial trait overlap persists, underscoring the insufficiency of morphology alone for resolving species boundaries and necessitating genomic validation.

Hierarchical clustering trees based on 22 morphological traits showed that ser. *P* clustered with *C. salicifolius*, consistent with the taxonomy system of Fryer and Hylmö (2009), where ser. *P* and ser. *S* together form sect. *Densiflori* T. T. Yu. Ser. *P* bifurcated into two subgroups: one containing *C. serotinus*, *C. glaucophyllus*, and *C. meiophyllus*, and the other encompassing remaining ser. *P* members (**Figure 4**). Ser. *B* clustered with *C.* sp. 3 and *C. marginatus*, and ser. *C* grouped with *C. microphyllus* (**Figure 4**). This topology underscores the polyphyletic nature of morphological series *P*, *B*, and *M*, revealing extensive phylogenetic discordance with traditional taxonomic groupings. Molecular phylogenies further complicated this picture: nuclear and cytoplasmic trees showed non-monophyly of ser. *P* but united *C. glaucophyllus* and *C. meiophyllus* (**Figures 5**, **6**), while chloroplast data failed to recover monophyletic series except for ser. *B* (excluding *C. insolitus* and *C. buxifolius* var. *vellaeus*). The 4D-site phylogeny dispersed ser. *B* taxa across three clades (**Figure 6**), highlighting profound discordance between morphological and molecular groupings.

These incongruences between morphological clustering and molecular phylogenies in the *C. buxifolius* complex and its closely related species likely stem from several evolutionary processes: hybridization, incomplete lineage sorting (ILS), and plastid capture. Hybridization erodes genetic boundaries, repositioning taxa phylogenetically toward parental lineages—a pattern observed in *C. cochleatus*, which clustered with putative parents in nuclear/cytoplasmic trees but aligned morphologically with 4D-site phylogeny (Liu et al., 2022). Such hybridization and subsequent gene flow promote morphological convergence, as observed in numerous plant species where introgression leads to overlapping morphological traits (Zheng et al., 2021; Wang et al., 2024). ILS allows ancestral genetic polymorphisms to persist during rapid speciation events, leading to discordance between gene and species trees (Feng et al., 2022). Plastid capture events, evidenced by the incongruence of mitochondrial and plastid phylogenies with nuclear phylogenies, further obscure evolutionary reconstruction (Liu et al., 2020; Yang et al., 2021). Environmental adaptation and phenotypic plasticity additionally modulate morphological expression (Zhang et al., 2024; Rosner and Morris, 2022; Karabourniotis et al., 2020), complicating the disentanglement of phylogenetic signals from ecological influences.

### 4.2 Species delimitation using an integrative approach

Interspecific hybridization serves as a pivotal mechanism in redefining species boundaries and restructuring systematic relationships across plant lineages (Harrison and Larson, 2014; Wang, 2017). Through reciprocal gene flow between phylogenetically distinct taxa, this process facilitates both the emergence of novel evolutionary lineages and the genomic remodeling of established species (Hörandl, 2022), progressively eroding conventional species demarcations and complicating our understanding of evolutionary history (Wang et al., 2021). Admixture analysis, a Bayesian clustering approach optimized for detecting contemporary hybridization signatures (Mattucci et al., 2019; Wang et al., 2019; Mao et al., 2017), was implemented to dissect the genetic architecture of the focal taxa. Cross-validation metrics identified *K* = 9 as the optimal genetic cluster configuration, resolving 14 species as a single gene pool indicative of evolutionary stability. These genomically cohesive clusters (ADMIXTURE ancestry ≥ 95%) formed the primary taxonomic hypotheses, which were subsequently subjected to a validation cascade requiring nuclear monophyly (SH-aLRT ≥ 80% and UFboot ≥ 95%), chloroplast lineage congruence, and morphological diagnosability (≥ 2 non-overlapping traits). These species are likely to represent recently established and stable taxa, with little evidence of recent hybridization events affecting their genetic composition. Conversely, admixed species exhibiting contributions from two or more gene pools revealed ongoing or historical hybridization processes, corroborating hybrid origins for these taxa. For such admixed lineages, taxonomic decisions relied on reconciling cytonuclear discordance (e.g., nuclear G2 dominance with chloroplast G3 affiliation in *C. marginatus*) and intermediate morphology, ultimately designating them as hybrids when all three lines of evidence concurred. This finding supported the hypothesis that hybridization has played a significant role in shaping the genetic diversity of these species.

The presence of admixed species complicates efforts to construct accurate phylogenetic trees, as hybridization can introduce conflicting signals into the data (Rieseberg and Soltis, 1991; Steenwyk et al., 2023). To mitigate these confounding effects, phylogenetic analyses frequently exclude recently hybridized taxa, prioritizing pure lineages to recover ancestral divergence patterns (McDade, 1992; Morales-Briones et al., 2018; Van Poucke et al., 2021; Yi et al., 2023). Our approach adopts this conservative philosophy but extends it to taxonomic practice: genomically admixed taxa (ADMIXTURE < 95%) were excluded from primary phylogenetic reconstruction but retained for hybrid designation if their cytonuclear–morphological profiles indicated stable reticulate origins. By focusing on non-recent hybrid species, it is possible to obtain a clearer picture of the evolutionary history and relationships among these taxa (McDade, 1992; Mallet, 2007). This conservative approach enhances phylogenetic accuracy by minimizing reticulate noise, thereby clarifying deep evolutionary relationships (Harrison and Larson, 2014; Mallet, 2007).

For taxonomic delineation within ser. *P*, ser. *B*, and related taxa, a hierarchical framework integrating multilocus evidence is proposed following Hong (2016) operational protocol for species delimitation. Nuclear monophyly (node support thresholds: SH-aLRT ≥ 80% and UFboot ≥ 95%) and stable genetic structure (ADMIXTURE cluster assignment probability ≥ 95%) constitute the primary criterion, as genome-wide SNPs provide a robust phylogenetic signal in resolving recent radiations (Joly et al., 2014). Morphological diagnosability (≥ 2 discontinuous traits) and chloroplast lineage congruence are required as secondary criteria to ensure taxonomic practicality and identify cytonuclear discordances from hybridization. The *K* = 9 admixture configuration provides the genomic baseline for initial species hypotheses, requiring subsequent validation through nuclear phylogenetic coherence and character-state divergence. This multilayered approach reconciles cytonuclear discordances while addressing the limitations of single-evidence taxonomic systems in hybrid-rich lineages, thereby circumventing the circularity risks inherent in criteria that rely on singular data types (Sukumaran and Knowles, 2017).

#### 4.2.1 Species representing the nine gene pools and their genetic relationship

In the nuclear phylogenetic tree constructed from 14 species representing nine gene pools, these species can be categorized into three distinct clades: NA, NB, and NC. In this study, we systematically investigate species delimitation and genetic relationships based on their respective positions within these three clades.

Clade NA serves as a paradigmatic example of species validation through integrated evidence derived from nuclear phylogeny and morphological differentiation. *C. conspicuus* and *C. sherriffii* (ser. *C*, G1) exhibit monophyly separately within clade NA (**Figure 10**). At *K* = 19, both *C. conspicuus* and *C. sherriffii* were found to share the same gene pool and cluster together in the PCA (**Figures 7**, **8**). Despite their genetic similarity, these two species display distinct ploidy levels: *C. conspicuus* is diploid, while *C. sherriffii* is triploid (Fryer and Hylmö, 2009). Although there exists cytonuclear discordance in *C. conspicuus* (**Figure 9**), both species demonstrate two or more discontinuous morphological traits that further support their distinction. *C. conspicuus* is characterized by its lack of lenticels on branches and has sparsely pilose adaxial leaf surfaces with elliptic to oblong-elliptic or oblong-ovate leaves featuring a callous-mucronate apex. In contrast, *C. sherriffii* displays prominent lenticels along with glabrous to subglabrous adaxial leaf surfaces; its leaves are elliptic to obovate with an obtuse or rotund apex. The monophyly observed in the nuclear phylogeny combined with significant morphological differentiation substantiates the recognition of *C. conspicuus* and *C. sherriffii* as distinct species.

While all four taxa within Clade NB are individually monophyletic, both *C. dammeri* and its subspecies *C. dammeri* subsp. *songmingensis* exhibit insufficient independent morphological characteristics to substantiate their taxonomic distinction. Morphologically, these two taxa display notable similarities, with the primary differences observed in the petiole length of sterile branches. Furthermore, they share an identical genetic structure (**Figures 7**, **8**) and present conflicting positions in nuclear versus plastid phylogenetic trees (*C. dammeri* is illustrated in **Figure 9**). However, given that only a single population was sampled for each taxon in this study, it remains inconclusive whether *C. dammeri* and *C. dammeri* subsp. *songmingensis* represent one or two distinct species. In contrast, *C. salicifolius* and *C. glaucophyllus* demonstrate clear divergence through independent gene pools (**Figure 7**), consistent positions in cytonuclear phylogenies (**Figure 9**), and complete morphological separation. *C. glaucophyllus* (ser. *P*) is characterized by nearly glabrous leaf abaxial surfaces, occasionally sparsely strigose hypanthium, and glabrous sepals, whereas *C. salicifolius* forms discrete clusters in morphological PCA (**Figure 3**).

Clade NC exhibits genomic–morphological congruence. The sister taxa, *C. fulvidus* (G5) and *C. serotinus* (G6), maintain nuclear monophyly while surpassing interspecific morphological thresholds through divergence in leaf texture (coriaceous vs. subcoriaceous), abaxial surface indumentum density (yellowish tomentose vs. subglabrous), and inflorescence architecture (compact vs. lax). Although *C. fulvidus* exhibits approximately 70% G5 ancestry in ADMIXTURE analysis, falling below the 95% genomic cohesion threshold, its recognition as a distinct species is unequivocally supported by two concordant lines of evidence. First, *C. fulvidus* (classified as *Cotoneaster hebephyllus* var. *fulvidus* W.W. Smith in the Catalogue of Life China, 2024 and the WFO Plant List: World Flora Online, 2024) is morphologically diagnosable from *C. hebephyllus* Diels by its evergreen habit (versus deciduous), coriaceous leaves (versus chartaceous), and initially indumented fruits (versus glabrous). Second, nuclear monophyly with maximal support (SH-aLRT = 100% and UFboot = 100%) and the phylogeny reconstructed by Meng et al. (2021) robustly separate *C. fulvidus* from *C. hebephyllus*. Importantly, these morphological and genetic boundaries align with the species delimitation criteria of Fryer and Hylmö (2009), demonstrating that the framework successfully validates taxonomic hypotheses even in cases of genomic ambiguity. This case exemplifies the framework’s capacity to reconcile genomic ambiguity through multilocus validation, where strong phylogenetic support and non-overlapping traits override subthreshold clustering signals. Similarly, *C. serotinus* has been treated as a variety of *C. glaucophyllus* in both the Catalogue of Life China (2024) and the WFO Plant List: World Flora Online (2024). Notably, *C. serotinus* can be distinguished from *C. glaucophyllus* by its dull adaxial leaves, elliptical or obovate leaf shapes, and short-acuminate apices. Together with genetic differentiation, these distinctions reinforce the classification of *C. serotinus* as a separate species (Fryer and Hylmö, 2009).

Hybrid interference in ser. *B* underscores the robustness of the framework. This grouping diverged from the original phylogenetic tree constructed using all 27 *Cotoneaster* species, indicating that the inclusion of hybrids may influence both the construction of the phylogenetic tree and the monophyly of ser. *B* (**Figure 9**). At *K* = 19, *C.* sp. 1 and *C. rockii* each exhibited distinct gene pools and were clearly distinguishable from one another in PCA, forming separate monophyletic clades within the phylogenetic trees (**Figures 7**, **8**, **10**). Morphologically, these species differ in traits such as adaxial lateral vein prominence (not obvious vs. more prominent), surface rugosity (absent vs. slightly present), and fruit indumentum (persistent vs. non-persistent), further reinforcing their classification as separate species. In this study, *C. buxifolius* was identified as a mixture of gene pools G6 and G7 (*K* = 9; **Figure 7**); therefore, classifying *C. rockii* as *C. buxifolius* var. *rockii* (G. Klotz) L.T. Lu and Brach based on Catalogue of Life China (2024) is not supported by our findings.

At last, three species of ser. *P*—*C. brickellii*, *C. turbinatus*, and *C. coriaceus*—each formed monophyletic groups with distinct gene pools (**Figures 7**, **10**). Morphologically, *C. brickellii* is distinguished from both *C. turbinatus* and *C. coriaceus* by its five to seven– lateral leaf veins (compared to 7–10) and shorter growth habit (1.5–2– versus 3–6 m). Additionally, *C. turbinatus* exhibits subtle divergence from *C. coriaceus*, characterized by subcoriaceous leaves as opposed to coriaceous ones and obovoid fruits. Based on congruent molecular monophyly, independent gene pools, and the presence of two or more diagnostic morphological characteristics, it is warranted that *C. brickellii*, *C. turbinatus*, and *C. coriaceus* be recognized as distinct species.

#### 4.2.2 Taxa with admixed genetic background and their phylogenetic relationship

##### 4.2.2.1 Taxa with complex genetic background in clade NA

*C. marginatus* exhibited a mosaic genomic architecture dominated by G2 (associated with *C. rockii*/*C.* sp. 1), with supplementary contributions from G1, G3, and G6 at *K* = 9, retaining residual admixture even at higher resolution (*K* = 19, **Figure 7**). Chloroplast phylogeny positioned this taxon within a clade containing *C. morrisonensis* and *C. dammeri* subsp. *songmingensis* (G3, **Figure 5**), contrasting with its morphological affinity to ser. *B* (G2, **Figure 4**). This cytonuclear discordance strongly suggests recent hybrid origins involving multiple parental lineages. A parallel pattern emerged in *C. buxifolius* var. *vellaeus*, which displayed genomic admixture from G1, G2, and G7. Admixture analysis implicated *C. rockii* (G2) as a likely progenitor, corroborated by morphological alignment with ser. *B*. However, plastid phylogeny revealed potential plastid capture from *C. microphyllus* (G1, **Figure 5**), while PCA positioned this variety intermediately between G1 and G2 clusters (**Figure 8**). Collectively, these findings indicate that both *C. marginatus* and *C. buxifolius* var. *vellaeus* arose through hybridization events involving *C. rockii* (G2) and additional unresolved parental taxa. Resolution of their evolutionary histories necessitates expanded population-level sampling to disentangle ancestral gene flow dynamics and identify cryptic contributors to their hybrid genomes.

*C. insolitus* primarily exhibited a G2 gene pool, with minor contributions from G1 and G6 at *K* = 9, indicating genetic admixture between *C.* sp. 1 (G2) and G1 at *K* = 19 (**Figure 7**). Phylogenetic analysis based on 4D sites revealed that it formed a monophyletic group alongside *C. conspicuus* and *C. sherriffii* (G1), while the chloroplast tree positioned it closest to *C. conspicuus* (**Figures 5**, **6**). Therefore, our data support the hypothesis that *C. insolitus* is a product of hybridization between *C. conspicuus* (G1) and *C.* sp. 1 (G2), although it may have also undergone introgression from other species. Its distinct morphological characteristics—such as the dark green adaxial leaf surface devoid of rugosity, persistent indumentum, and slightly depressed lateral veins—contradict its varietal designation as *C. buxifolius* var. *rockii* in the Catalogue of Life China (2024).

##### 4.2.2.2 Taxa with complex genetic background in clade NB

*C.* sp. 2 exemplifies the challenges of reconciling conflicting evolutionary signals: nuclear data affiliate it with *C. serotinus* (G5), plastid phylogeny links it to *C. turbinatus* (GS population), and morphological clustering aligns it with *C. salicifolius* (G3; **Figures 4**–**6**). This tripartite discordance underscores the limitations of single-evidence approaches in resolving complex hybrid taxa and highlights the challenges in precisely determining their taxonomic status.

*C. morrisonensis* and *C. cochleatus* likely originated from hybridization between *C. microphyllus* and *C. dammeri* lineages. Chloroplast phylogeny resolved *C. morrisonensis* (G3-dominated), forming a monophyletic group with *C. dammeri* subsp. *songmingensis* (G3), whereas *C. cochleatus* (G1-dominated) clustered with *C. microphyllus* (G1) (**Figure 5**). Morphologically, *C. cochleatus* exhibits close affinity to *C. microphyllus* in leaf architecture while sharing the rooting habit of *C. dammeri*. The combined evidence of genomic admixture, cytonuclear discordance, and morphological intermediacy collectively demonstrates the hybrid origin hypothesis for these taxa.

*C. meiophyllus* (with G4 and G6 genetic admixture) clustered morphologically and in chloroplast phylogeny with *C. glaucophyllus* (G4), supporting its taxonomic recognition as *C. glaucophyllus* var. *meiophyllus* W. W. Smith, which is consistent with the taxonomic treatments in both the Catalogue of Life China (2024) and the WFO Plant List: World Flora Online (2024). Conversely, *C. buxifolius* and *C. lidjiangensis* (G6 and G7 admixture) showed identical gene pools and failed to form distinct phylogenetic branches (**Figure 7**), with no diagnostic morphological differences observed. Minor variations in stamen number and inflorescence size fell within the normal range of *C. buxifolius*, supporting the taxonomic treatment that *C. lidjiangensis* was synonymized with *C. buxifolius* (Catalogue of Life China, 2024).

*C. argenteus*, exhibiting a nuclear genomic admixture of G4 (*C. glaucophyllus*) and G7 (*C. delavayanus*), meets the primary species delimitation criterion that it constituted a monophyletic taxon in nuclear phylogeny analyses (**Figure 6**). While chloroplast data place it within the *C. rockii* clade (**Figure 5**), this discordance is secondary to the nuclear evidence under the proposed framework. Morphologically clustering, *C. argenteus* is close to *C. rockii* and *C. delavayanus*, differing slightly in the adaxial leaf surface, which is dull or slightly shiny, and in the lightly impressed lateral veins, which lack prominent characteristics. Further investigation is needed. However, its consistent differentiation from *C. buxifolius* via elliptic or obovate leaves (cuneate base), <10-mm fertile shoots, and 1–3-flowered inflorescences—coupled with sufficient genetic and phylogenetic distinction—warrants species-level recognition, rejecting its proposed synonymy of *C. buxifolius* (Catalogue of Life China, 2024; WFO Plant List: World Flora Online, 2024).

##### 4.2.2.3 Taxa with complex genetic background in clade NC

The taxonomic status of *C.* sp. 3 and *C.* sp. 4 remained unresolved under the hierarchical framework. Both taxa exhibited nuclear monophyly in the 4D-sites phylogeny (**Figure 6**), fulfilling the primary criterion, but their admixed genomic compositions (*C.* sp. 4 G8-dominated with G4 and G3 contributions; *C.* sp. 3: G8–G3–G7 admixture; **Figure 7**) and conflicting morphological clustering (*C.* sp. 4 with *C. coriaceus* and *C. lacteus*; *C.* sp. 3 with ser. *B* taxa; **Figure 4**) precluded definitive delimitation. Notably, their chloroplast genomes formed a strongly supported clade with *C. brickellii* and *C. pannosus* (**Figure 5**), suggesting ancestral hybridization or incomplete lineage sorting as potential drivers of discordance.

*C. pannosus* exemplified multilayered taxonomic ambiguity. The admixed nuclear genomes (G7–G8 ancestry; YHC population with additional introgression) aligned with its dual phylogenetic placements: the JSD population clustered with *C. delavayanus* (4D-site tree), while the YHC population grouped with *C. brickellii* (**Figure 6**). Chloroplast data further complicated this pattern, with one population aligning with *C. brickellii* (WS population) and another with *C. turbinatus*, *C. coriaceus*, and *C. brickellii* (**Figure 5**). Although population-level nuclear monophyly and cytonuclear discordance supported hybrid origins between *C. delavayanus* and *C. brickellii*, lineage-wide polyphyly precluded formal species validation under the framework’s criteria.

*C. lacteus* displays conflicting evolutionary signals across datasets. Genomic admixture analysis at multiple resolutions (*K* = 9–19) identified ancestral contributions from four gene pools: G9 (*C. coriaceus*), G7, G3, and G4 (**Figure 7**). Nuclear phylogeny confirms its distinct evolutionary lineage through monophyly in 4D-site analysis (**Figure 6**), meeting the primary criterion for species recognition. However, chloroplast phylogeny groups it with *C. coriaceus* (**Figure 5**), and similarity to this species has led to its current synonymy under this species (Catalogue of Life China, 2024; WFO Plant List: World Flora Online, 2024). This conflict between nuclear independence and plastid/morphological alignment may stem from multiple evolutionary mechanisms. Hybridization scenarios could explain the pattern if *C. coriaceus* served as the maternal progenitor, contributing both chloroplast DNA and key morphological traits, while nuclear genomes diverged through subsequent backcrossing (Liu et al., 2022). Alternatively, recent divergence from *C. coriaceus* may account for shared plastid haplotypes and morphology if insufficient time has elapsed for complete lineage sorting (Pillon et al., 2007). A third possibility involves ILS of ancestral polymorphisms, where stochastic retention of plastid haplotypes persists despite nuclear differentiation (Feng et al., 2022). Distinguishing these scenarios will require population-level genomic investigations to quantify introgression patterns and assess lineage sorting dynamics across its distribution range.

## 5 Conclusion

In this paper, we delineate complex species groups comprising up to 27 species through population genomics analysis. Our findings reveal that 14 of these species represent nine distinct gene pools, while the remaining 13 species have arisen from hybridization events involving these primary taxa. This study provides robust support for future revisions of the taxonomic classification of these species. However, our work is not yet exhaustive. Limited sampling may result in the omission of parental species, thereby affecting the accuracy of hybrid origin determination. This research offers a reliable framework for subsequent studies, underscoring the importance of expanding both species and population sampling to more accurately elucidate the hybrid origins of *Cotoneaster* species and specific taxa.

## Data Availability

The raw ddRAD-seq data for all *Cotoneaster* taxa generated for this study was uploaded to GenBank under the BioProject PRJNA1196588.

## Supplementary material

Supplementary Figure 1 Cyto-nuclear conflicts among two clades in *Cotoneaster* by comparison of chloroplast genome tree (left) and species tree (right) (Meng et al., 2021). Bayesian posterior probabilities (BIPP) are 1.00 and Ultrafast bootstrap support values (UFBS) are ≥95, unless otherwise indicated (left). Local posterior probabilities (Astral-PP) are ≥0.7 and multilocus bootstrap values (Astral-BS) are ≥70%, unless otherwise indicated (right). The red boxes highlight *C*. ser. *Pannosi*, *C*. ser. *Buxifolii* and related taxa.

Supplementary Figure 2 Contribution of variables to dim 1 and dim 2 in PCA based on 22 traits.

Supplementary Figure 3 Variation of Cross-Validation error with number of ancestral populations (*K*) in admixture analysis based on unlinked 4D sites.

## References

[B1] Alexander D. H. Novembre J. Lange K. (2009). Fast model-based estimation of ancestry in unrelated individuals. Genome Res. 19, 1655–1664. doi: 10.1101/gr.094052.109 19648217 PMC2752134

[B2] Andrews S. (2010). FastQC: a quality control tool for high throughput sequence data. Anal. Biochem. 548, 38–43. doi: 10.1016/j.ab.2018.01.028 29410015

[B3] Anjos M. S. Bitencourt J. A. Nunes L. A. Sarmento-Soares L. M. Carvalho D. C. Armbruster J. W.. (2020). Species delimitation based on integrative approach suggests reallocation of genus in Hypostomini catfish (Siluriformes, Loricariidae). Hydrobiologia 847, 563–578. doi: 10.1007/s10750-019-04121-z

[B4] Burgess M. B. Cushman K. R. Doucette E. T. Talent N. Frye C. T. Campbell C. S. (2014). Effects of apomixis and polyploidy on diversification and geographic distribution in Amelanchier (Rosaceae). Am. J. Bot. 101, 1375–1387. doi: 10.3732/ajb.1400113 25156985

[B5] Campbell C. S. Dickinson T. A. (1990). Apomixis, patterns of morphological variation, and species concepts in subfam. Maloideae (Rosaceae). Syst. Bot. 15, 124–135. doi: 10.2307/2419022

[B6] Campbell C. S. Evans R. C. Morgan D. R. Dickinson T. A. Arsenault M. P. (2007). Phylogeny of subtribe Pyrinae (formerly the Maloideae, Rosaceae): Limited resolution of a complex evolutionary history. Plant Syst. Evol. 266, 119–145. doi: 10.1007/s00606-007-0545-y

[B7] Campbell C. S. Greene C. W. Dickinson T. A. (1991). Reproductive biology in subfam. Maloideae (Rosaceae). Syst. Bot. 16, 333–349. doi: 10.2307/2419284

[B8] Castresana J. (2000). Selection of conserved blocks from multiple alignments for their use in phylogenetic analysis. Mol. Biol. Evol. 17, 540–552. doi: 10.1093/oxfordjournals.molbev.a026334 10742046

[B9] Catalogue of Life China (2024). CoLChina. Available online at: http://www.sp2000.org.cn/CoLChina (Accessed January 25, 2025).

[B10] Chen S. Zhou Y. Chen Y. Gu J. (2018). fastp: an ultra-fast all-in-one FASTQ preprocessor. Bioinformatics 34, i884–i890. doi: 10.1093/bioinformatics/bty560 30423086 PMC6129281

[B11] Cushman K. R. Burgess M. B. Doucette E. T. Nelson G. A. Campbell C. S. (2017). Species delimitation in tetraploid, apomictic Amelanchier (Rosaceae). Syst. Bot. 42, 234–256. doi: 10.1600/036364417X695529

[B12] Danecek P. Bonfield J. K. Liddle J. Marshall J. Ohan V. Pollard M. O.. (2021). Twelve years of SAMtools and BCFtools. Gigascience 10, giab008. doi: 10.1093/gigascience/giab008 33590861 PMC7931819

[B13] Dickinson T. A. (2018). Sex and rosaceae apomicts. Taxon 67, 1093–1107. doi: 10.12705/676.7

[B14] Dickinson T. Lo E. Talent N. (2007). Polyploidy, reproductive biology, and Rosaceae: understanding evolution and making classifications. Plant Syst. Evol. 266, 59–78. doi: 10.1007/s00606-007-0541-2

[B15] Doyle J. J. Doyle J. L. (1987). A rapid DNA isolation procedure for small quantities of fresh leaf tissue. Phytochemical Bull. 19, 11–15.

[B16] Ely C. V. Andrade B. O. Iganci J. R. V. Boldrini I. I. (2018). Integrative taxonomy improves delimitation in Hypericum subspecies. Perspect. Plant Ecol. Evol. Syst. 34, 68–76. doi: 10.1016/j.ppees.2018.08.005

[B17] Ewels P. Magnusson M. Lundin S. Käller M. (2016). MultiQC: summarize analysis results for multiple tools and samples in a single report. Bioinformatics 32, 3047–3048. doi: 10.1093/bioinformatics/btw354 27312411 PMC5039924

[B18] Feng S. Bai M. Rivas-González I. Li C. Liu S. Tong Y.. (2022). Incomplete lineage sorting and phenotypic evolution in marsupials. Cell 185, 1646–1660.e18. doi: 10.1016/j.cell.2022.03.034 35447073 PMC9200472

[B19] Flinck K. E. Hylmö B. (1966). A list of series and species in genus *cotoneaster* . Botaniska Notiser 119, 445–462.

[B20] Francis R. (2017). pophelper: an R package and web app to analyse and visualize population structure. Mol. Ecol. Resour. 17, 27–32. doi: 10.1111/1755-0998.12509 26850166

[B21] Fryer J. Hylmö B. (2009). Cotoneasters: A Comprehensive Guide to Shrubs for Flowers, Fruit, and Foliage (Portland and London: Timber Press).

[B22] Gower J. C. (1971). A general coefficient of similarity and some of its properties. Biometrics 27, 857–871. doi: 10.2307/2528823

[B23] Harrison R. G. Larson E. L. (2014). Hybridization, introgression, and the nature of species boundaries. J. Heredity 105, 795–809. doi: 10.1093/jhered/esu033 25149255

[B24] Hodel R. G. Zimmer E. A. Liu B. B. Wen J. (2022). Synthesis of nuclear and chloroplast data combined with network analyses supports the polyploid origin of the apple tribe and the hybrid origin of the Maleae—Gillenieae clade. Front. Plant Sci. 12. doi: 10.3389/fpls.2021.820997 PMC8822239 35145537

[B25] Hong D.-Y. (2016). Biodiversity pursuits need a scientific and operative species concept. Biodiversity Sci. 24, 979. doi: 10.17520/biods.2016203

[B26] Hörandl E. (2022). Novel approaches for species concepts and delimitation in polyploids and hybrids. Plants 11, 204. doi: 10.3390/plants11020204 35050093 PMC8781807

[B27] Hunter J. (2007). Matplotlib: A 2D graphics environment. Comput. Sci. Eng. 9, 90–95. doi: 10.1109/mcse.2007.55

[B28] Jin J. J. Yu W. B. Yang J. B. Song Y. DePamphilis C. W. Yi T. S.. (2020). GetOrganelle: a fast and versatile toolkit for accurate *de novo* assembly of organelle genomes. Genome Biol. 21, 1–31. doi: 10.1186/s13059-020-02154-5 PMC7488116 32912315

[B29] Joly S. Hipp A. L. Eaton D. A. R. Cavender-Bares J. Fitzek E. Nipper R.. (2014). A framework phylogeny of the american oak clade based on sequenced RAD data. PloS One 9, e93975. doi: 10.1371/journal.pone.0093975 24705617 PMC3976371

[B30] Kalyaanamoorthy S. Minh B. Q. Wong T. K. Von Haeseler A. Jermiin L. S. (2017). ModelFinder: fast model selection for accurate phylogenetic estimates. Nat. Methods 14, 587–589. doi: 10.1038/nmeth.4285 28481363 PMC5453245

[B31] Karabourniotis G. Liakopoulos G. Nikolopoulos D. Bresta P. (2020). Protective and defensive roles of non-glandular trichomes against multiple stresses: structure–function coordination. J. For. Res. 31, 1–12. doi: 10.1007/s11676-019-01034-4

[B32] Katoh K. Standley D. M. (2013). MAFFT multiple sequence alignment software version 7: improvements in performance and usability. Mol. Biol. Evol. 30, 772–780. doi: 10.1093/molbev/mst010 23329690 PMC3603318

[B33] Koehne E. (1893). Deutsche Dendrologie. Stuttgart: Verlag von Ferdinand Enke.

[B34] Lê S. Josse J. Husson F. (2008). FactoMineR: an R package for multivariate analysis. J. Stat. Software 25, 1–18. doi: 10.18637/jss.v025.i01

[B35] Li H. (2013). Aligning sequence reads, clone sequences and assembly contigs with BWA-MEM. arXiv preprint arXiv:1303.3997. doi: 10.48550/arXiv.1303.3997

[B36] Li F. Fan Q. Li Q. Chen S. Guo W. Cui D.. (2014). Molecular phylogeny of Cotoneaster (Rosaceae). inferred from nuclear ITS and multiple chloroplast sequences. Plant Syst. Evol. 300, 1533–1546. doi: 10.1007/s00606-014-0980-5

[B37] Li M. Chen S. Zhou R. Fan Q. Li F. Liao W. (2017). Molecular evidence for natural hybridization between *Cotoneaster dielsianus* and *C. glaucophyllus* . Front. Plant Sci. 8, 704. doi: 10.3389/fpls.2017.00704 28536587 PMC5422516

[B38] Li Y. C. Wen J. Ren Y. Zhang J. Q. (2019). From seven to three: Integrative species delimitation supports major reduction in species number in Rhodiola section Trifida (Crassulaceae) on the Qinghai-Tibetan Plateau. Taxon 68, 268–279. doi: 10.1002/tax.12052

[B39] Linan A. G. Lowry P. P. Miller A. J. Schatz G. E. Sevathian J. C. Edwards C. E. (2021). RAD-sequencing reveals patterns of diversification and hybridization, and the accumulation of reproductive isolation in a clade of partially sympatric, tropical island trees. Mol. Ecol. 30, 4520–4537. doi: 10.1111/mec.15736 33210759

[B40] Liston A. Weitemier K. A. Letelier L. Podani J. Zong Y. Liu L.. (2021). Phylogeny of Crataegus (Rosaceae) based on 257 nuclear loci and chloroplast genomes: evaluating the impact of hybridization. PeerJ 9, e12418. doi: 10.7717/peerj.12418 34754629 PMC8555502

[B41] Liu J. Q. (2016). The integrative species concept” and “species on the speciation way. Biodivers. Sci. 24, 1004–1008. doi: 10.17520/biods.2016222

[B42] Liu L. X. Du Y. X. Folk R. A. Wang S. Y. Soltis D. E. Shang F. D.. (2020). Plastome evolution in Saxifragaceae and multiple plastid capture events involving Heuchera and Tiarella. Front. Plant Sci. 11. doi: 10.3389/fpls.2020.00361 PMC7193090 32391025

[B43] Liu B. B. Ren C. Kwak M. Hodel R. G. Xu C. He J.. (2022). Phylogenomic conflict analyses in the apple genus Malus sl reveal widespread hybridization and allopolyploidy driving diversification, with insights into the complex biogeographic history in the Northern Hemisphere. J. Integr. Plant Biol. 64, 1020–1043. doi: 10.1111/jipb.13246 35274452

[B44] Liu S. Zhang L. Sang Y. Lai Q. Zhang X. Jia C.. (2022). Demographic history and natural selection shape patterns of deleterious mutation load and barriers to introgression across Populus genome. Mol. Biol. Evol. 39, msac008. doi: 10.1093/molbev/msac008 35022759 PMC8826634

[B45] Lu L. D. Brach A. R. (2003). “Cotoneaster,” in Flora of China, vol. 9. (Beijing: Science Press; St. Louis: Missouri Botanical Garden Press), 85–108.

[B46] Macková L. (2020). Microevolutionary processes in selected genera of the Rosaceae family. Charles University, Faculty of Science, Department of Botany, Prague (CZ).

[B47] Maharachchikumbura S. S. Chen Y. Ariyawansa H. A. Hyde K. D. Haelewaters D. Perera R. H.. (2021). Integrative approaches for species delimitation in Ascomycota. Fungal Divers. 109, 155–179. doi: 10.1007/s13225-021-00486-6

[B48] Mallet J. (2007). Hybrid speciation. Nature 446, 279–283. doi: 10.1038/nature05706 17361174

[B49] Mao J.-F. Ma Y. Zhou R. (2017). Approaches used to detect and test hybridization: combining phylogenetic and population genetic analyses. Biodivers. Sci. 25, 577–599. doi: 10.17520/biods.2017097

[B50] Marçais G. Delcher A. L. Phillippy A. M. Coston R. Salzberg S. L. Zimin A. (2018). MUMmer4: A fast and versatile genome alignment system. PloS Comput. Biol. 14, e1005944. doi: 10.1371/journal.pcbi.1005944 29373581 PMC5802927

[B51] Martin M. (2011). Cutadapt removes adapter sequences from high-throughput sequencing reads. EMBnet J. 17, 10–12. doi: 10.14806/ej.17.1.200

[B52] Mattucci F. Galaverni M. Lyons L. A. Alves P. C. Randi E. Velli E.. (2019). Genomic approaches to identify hybrids and estimate admixture times in European wildcat populations. Sci. Rep. 9, 11612. doi: 10.1038/s41598-019-48002-w 31406125 PMC6691104

[B53] McDade L. A. (1992). Hybrids and phylogenetic systematics II. The impact of hybrids on cladistic analysis. Evolution 46, 1329–1346. doi: 10.1111/j.1558-5646.1992.tb01127.x 28569006

[B54] McKenna A. Hanna M. Banks E. Sivachenko A. Cibulskis K. Kernytsky A.. (2010). The Genome Analysis Toolkit: a MapReduce framework for analyzing next-generation DNA sequencing data. Genome Res. 20, 1297–1303. doi: 10.1101/gr.107524.110 20644199 PMC2928508

[B55] Meng K. K. Chen S. F. Xu K. W. Zhou R. C. Li M. W. Dhamala M. K.. (2021). Phylogenomic analyses based on genome-skimming data reveal cyto-nuclear discordance in the evolutionary history of Cotoneaster (Rosaceae). Mol. Phylogenet. Evol. 158, 107083. doi: 10.1016/j.ympev.2021.107083 33516804

[B56] Meng K. K. Liao W. B. Wei S. Chen S. Li M. Ma Y.. (2024). Chromosome-scale genome assembly and annotation of *Cotoneaster glaucophyllus* . Sci. Data 11, 406. doi: 10.1038/s41597-024-03246-8 38649372 PMC11035681

[B57] Morales-Briones D. F. Liston A. Tank D. C. (2018). Phylogenomic analyses reveal a deep history of hybridization and polyploidy in the Neotropical genus Lachemilla (Rosaceae). New Phytol. 218, 1668–1684. doi: 10.1111/nph.15099 29604235

[B58] Nguyen L. T. Schmidt H. A. Von Haeseler A. Minh B. Q. (2015). IQ-TREE: a fast and effective stochastic algorithm for estimating maximum-likelihood phylogenies. Mol. Biol. Evol. 32, 268–274. doi: 10.1093/molbev/msu300 25371430 PMC4271533

[B59] Pillon Y. Fay M. F. Hedrén M. Bateman R. M. Devey D. S. Shipunov A. B.. (2007). Evolution and temporal diversification of western European polyploid species complexes in Dactylorhiza (Orchidaceae). Taxon 56, 1185–1208. doi: 10.2307/25065911

[B60] Potter D. Eriksson T. Evans R. C. Oh S. Smedmark J. E. E. Morgan D. R.. (2007). Phylogeny and classification of rosaceae. Plant Syst. Evol. 266, 5–43. doi: 10.1007/s00606-007-0539-9

[B61] Prebus M. M. (2021). Phylogenomic species delimitation in the ants of the Temnothorax salvini group (Hymenoptera: Formicidae): an integrative approach. Syst. Entomol. 46, 307–326. doi: 10.1111/syen.12463

[B62] Purcell S. Neale B. Todd-Brown K. Thomas L. Ferreira M. A. Bender D.. (2007). PLINK: a tool set for whole-genome association and population-based linkage analyses. Am. J. Hum. Genet. 81, 559–575. doi: 10.1086/519795 17701901 PMC1950838

[B63] Renaut S. Rowe H. C. Ungerer M. C. Rieseberg L. H. (2014). Genomics of homoploid hybrid speciation: diversity and transcriptional activity of long terminal repeat retrotransposons in hybrid sunflowers. Philos. Trans. R. Soc. B: Biol. Sci. 369, 20130345. doi: 10.1098/rstb.2013.0345 PMC4071519 24958919

[B64] Rieseberg L. H. Soltis D. (1991). Phylogenetic consequences of cytoplasmic gene flow in plants. Evolutionary Trends Plants 5, 65–84.

[B65] Rosner S. Morris H. (2022). Breathing life into trees: the physiological and biomechanical functions of lenticels. IAWA J. 43, 234–262. doi: 10.1163/22941932-bja10090

[B66] Rothleutner J. J. Friddle M. W. Contreras R. N. (2016). Ploidy levels, relative genome sizes, and base pair composition in Cotoneaster. J. Am. Soc. Hortic. Sci. 141, 457–466. doi: 10.21273/JASHS03776-16

[B67] Steenwyk J. L. Li Y. Zhou X. Shen X.-X. Rokas A. (2023). Incongruence in the phylogenomics era. Nat. Rev. Genet. 24, 834–850. doi: 10.1038/s41576-023-00620-x 37369847 PMC11499941

[B68] Sukumaran J. Knowles L. L. (2017). Multispecies coalescent delimits structure, not species. Proc. Natl. Acad. Sci. U S A 114, 1607–1612. doi: 10.1073/pnas.1607921114 28137871 PMC5320999

[B69] Sun J. Zhao D. Qiao P. Wang Y. Wu P. Wang K.. (2024). Phylogeny of genera in Maleae (Rosaceae) based on chloroplast genome analysis. Front. Plant Sci. 15. doi: 10.3389/fpls.2024.1367645 PMC11002139 38595768

[B70] Van Poucke K. Haegeman A. Goedefroit T. Focquet F. Leus L. Jung M. H.. (2021). Unravelling hybridization in Phytophthora using phylogenomics and genome size estimation. IMA fungus 12, 1–24. doi: 10.1186/s43008-021-00068-w 34193315 PMC8246709

[B71] Virtanen P. Gommers R. Oliphant T. E. Haberland M. Reddy T. Cournapeau D.. (2020). SciPy 1.0: fundamental algorithms for scientific computing in Python. Nat. Methods 17, 261–272. doi: 10.1038/s41592-019-0686-2 32015543 PMC7056644

[B72] Volk G. M. Henk A. D. Richards C. M. Bassil N. Postman J. (2019). Chloroplast sequence data differentiate Maleae, and specifically Pyrus, species in the USDA-ARS National Plant Germplasm System. Genet. Resour. Crop Evol. 66, 5–15. doi: 10.1007/s10722-018-0691-9

[B73] Wagner N. D. He L. Hörandl E. (2020). Phylogenomic relationships and evolution of polyploid Salix species revealed by RAD sequencing data. Front. Plant Sci. 11. doi: 10.3389/fpls.2020.01077 PMC7379873 32765560

[B74] Walker B. J. Abeel T. Shea T. Priest M. Abouelliel A. Sakthikumar S.. (2014). Pilon: an integrated tool for comprehensive microbial variant detection and genome assembly improvement. PloS One 9, e112963. doi: 10.1371/journal.pone.0112963 25409509 PMC4237348

[B75] Wang Y. (2017). Natural hybridization and speciation. Biodivers. Sci. 25, 565–576. doi: 10.17520/biods.2017041

[B76] Wang Z. Jiang Y. Bi H. Lu Z. Ma Y. Yang X.. (2021). Hybrid speciation via inheritance of alternate alleles of parental isolating genes. Mol. Plant 14, 208–222. doi: 10.1016/j.molp.2020.11.008 33220509

[B77] Wang H. Li X. Y. Jiang Y. Jin Z. T. Ma D. K. Liu B.. (2024). Refining the phylogeny and taxonomy of the apple tribe Maleae (Rosaceae): insights from phylogenomic analyses of 563 plastomes and a taxonomic synopsis of Photinia and its allies in the Old World. Phytokeys 242, 161–227. doi: 10.3897/phytokeys.242.117481 38854497 PMC11161682

[B78] Wang X. Liao S. Zhang Z. Zhang J. Mei L. Li H. (2024). Hybridization, polyploidization, and morphological convergence make dozens of taxa into one chaotic genetic pool: a phylogenomic case of the *Ficus erecta* species complex (Moraceae). Front. Plant Sci. 15. doi: 10.3389/fpls.2024.1354812 PMC11002808 38595762

[B79] Wang D. Wang Z. Kang X. Zhang J. (2019). Genetic analysis of admixture and hybrid patterns of Populus hopeiensis and P. tomentosa. Sci. Rep. 9, 4821. doi: 10.1038/s41598-019-41320-z 30886279 PMC6423230

[B80] WFO Plant List: World Flora Online (2024). Available online at: http://www.worldfloraonline.org (Accessed January 25, 2025).

[B81] Yang Q. Fu Y. Deng Q. Wang Y. Tao L. Liu L.. (2010). A method for excellent quality DNA extraction of loquat. North. Hort 20, 134–137.

[B82] Yang Y. Y. Qu X. J. Zhang R. Stull G. W. Yi T. S. (2021). Plastid phylogenomic analyses of Fagales reveal signatures of conflict and ancient chloroplast capture. Mol. Phylogenet. Evol. 163, 107232. doi: 10.1016/j.ympev.2021.107232 34129935

[B83] Yi H. Dong S. Yang L. Wang J. Kidner C. Kang M. (2023). Genome-wide data reveal cryptic diversity and hybridization in a group of tree ferns. Mol. Phylogenet. Evol. 184, 107801. doi: 10.1016/j.ympev.2023.107801 37088242

[B84] Yu D. J. Lu L. D. Gu C. Z. (1974). “Cotoneaster,” in Flora of China, vol. 36. (Science Press, Beijing), 107–178.

[B85] Zhang K. L. Leng Y. N. Hao R. R. Zhang W. Y. Li H. F. Chen M. X.. (2024). Adaptation of high-altitude plants to harsh environments: application of phenotypic-variation-related methods and multi-omics techniques. Int. J. Mol. Sci. 25, 12666. doi: 10.3390/ijms252312666 39684378 PMC11641277

[B86] Zheng W. Yan L. J. Burgess K. S. Luo Y. H. Zou J. Y. Qin H. T.. (2021). Natural hybridization among three *Rhododendron* species (Ericaceae) revealed by morphological and genomic evidence. BMC Plant Biol. 21, 1–12. doi: 10.1186/s12870-021-03312-y 34763662 PMC8582147

[B87] Zhou L. H. Wu Z. Y. (2001). Taxonomic revision of the series *Buxifolii* in *Cotoneaster* (Rosaceae). Acta Botanica Yunnanica 23, 29–36.

