# Supplementary figures and images for: Phylogenomic relationships and species delimitation of Cotoneaster ser. Pannosi, ser. Buxifolii, and related taxa
Source: Front Plant Sci. 2025 May 20;16:1575925. doi: 10.3389/fpls.2025.1575925 (PMC12129942; doi:10.3389/fpls.2025.1575925)

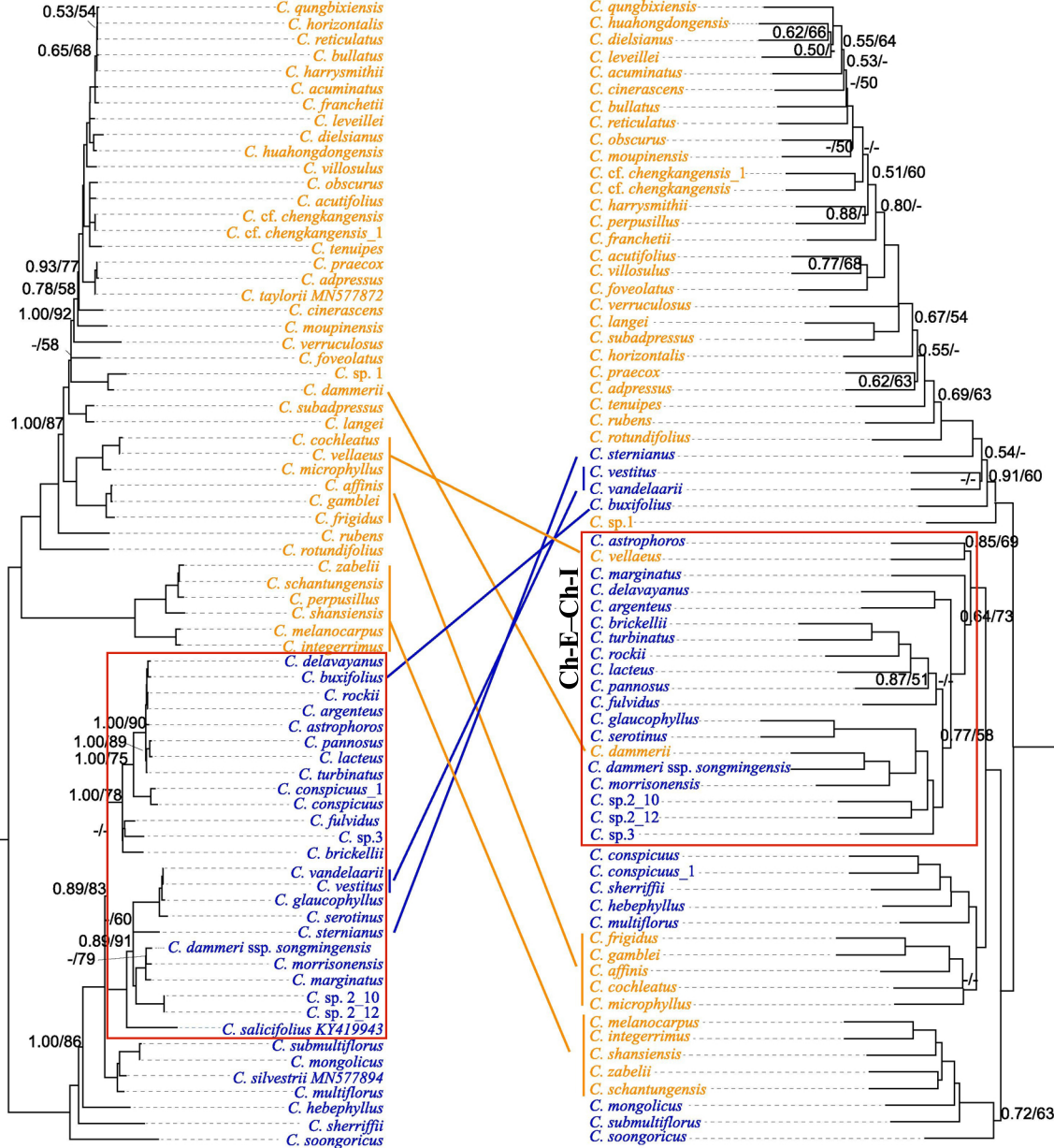

6.0E-4

0.9

Supplement: Supplementary Figure 1 — Cyto-nuclear conflicts among two clades in Cotoneaster by comparison of chloroplast genome tree (left) and species tree (right) (Meng et al., 2021). Bayesian posterior probabilities (BIPP) are 1.00 and Ultrafast bootstrap support values (UFBS) are ≥95, unless otherwise indicated (left). Local posterior probabilities (Astral-PP) are ≥0.7 and multilocus bootstrap values (Astral-BS) are ≥70%, unless otherwise indicated (right). The red boxes highlight C. ser. Pannosi, C. ser. Buxifolii and related taxa. [file DataSheet1.pdf]

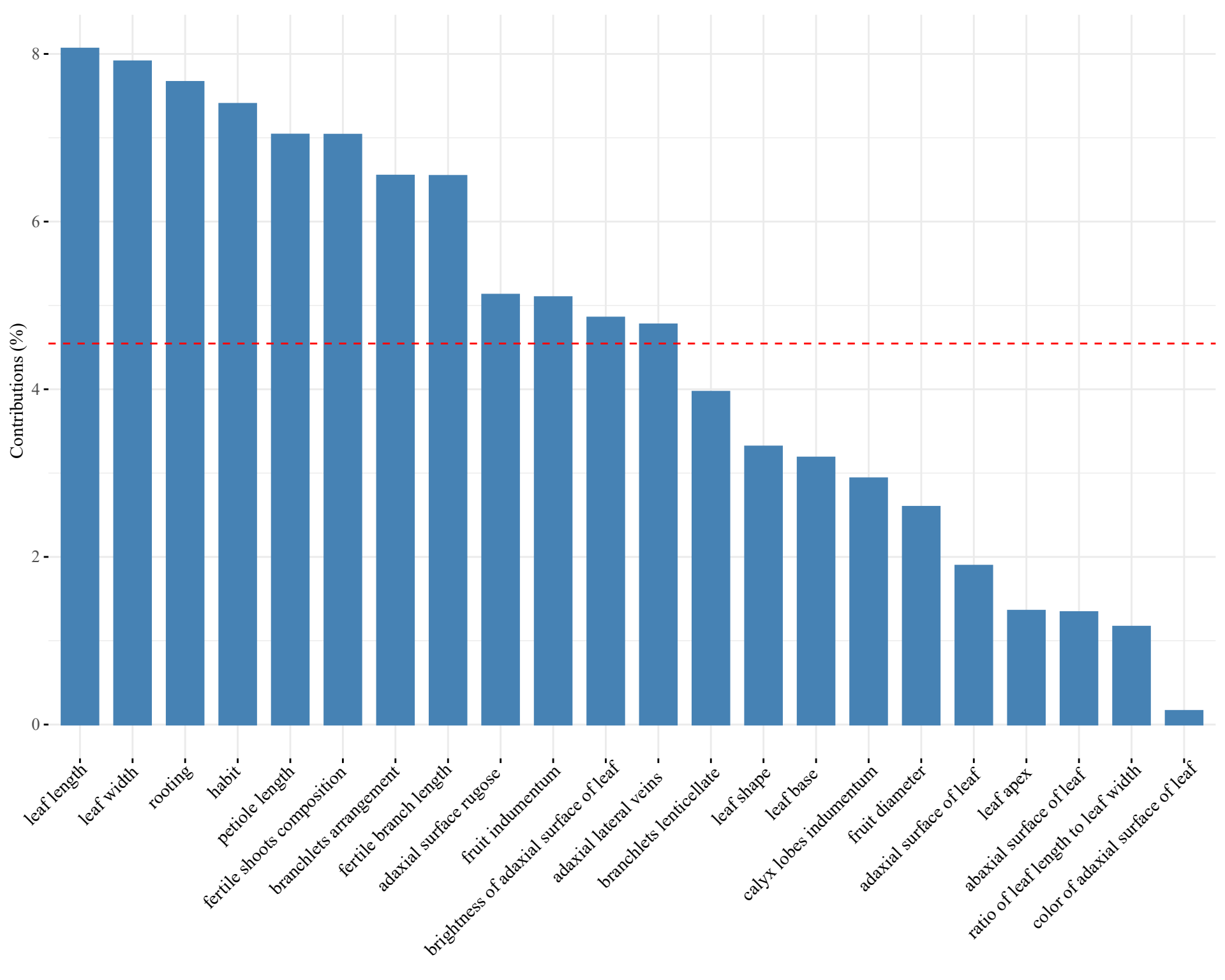

Supplement: Supplementary Figure 2 — Contribution of variables to dim 1 and dim 2 in PCA based on 22 traits. [file DataSheet2.pdf]

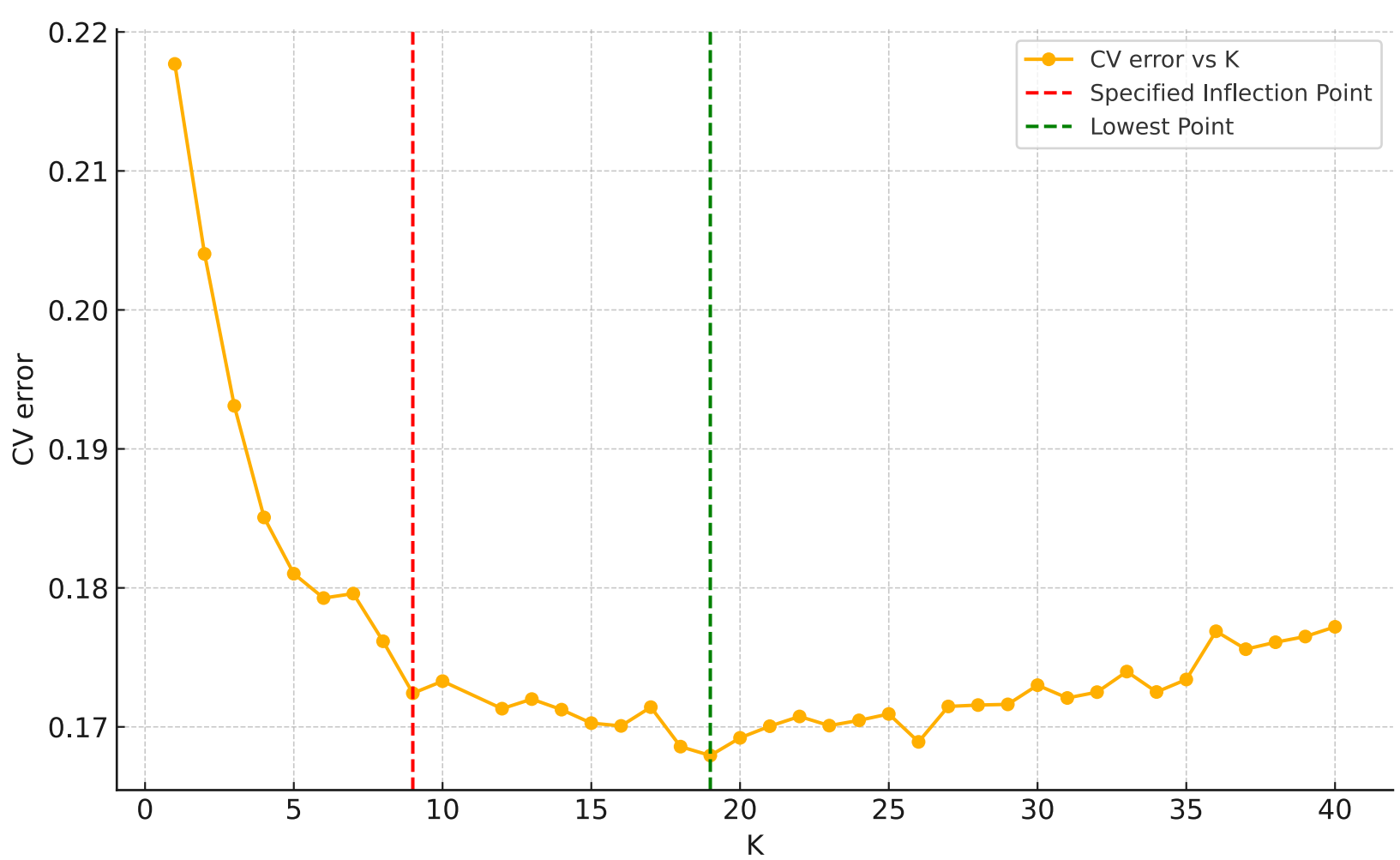

Supplement: Supplementary Figure 3 — Variation of Cross-Validation error with number of ancestral populations (K) in admixture analysis based on unlinked 4D sites. [file DataSheet3.pdf]
